# A set of cancer stem cell homing peptides associating with the glycan moieties of glycosphingolipids

**DOI:** 10.18632/oncotarget.24960

**Published:** 2018-04-17

**Authors:** Yu-Hsiu Su, Tai-Yun Lin, Hung-Jen Liu, Chin-Kai Chuang

**Affiliations:** ^1^ Division of Biotechnology, Animal Technology Laboratories, Agricultural Technology Research Institute, Hsinchu City 30093, Taiwan; ^2^ Institute of Molecular Biology, National Chung Hsing University, Taichung City 40227, Taiwan; ^3^ Rong Hsing Research Center for Translational Medicine, National Chung Hsing University, Taichung City 40227, Taiwan; ^4^ The iEGG and Animal Biotechnology Center, National Chung Hsing University, Taichung City 40227, Taiwan

**Keywords:** cancer stem cells, homing peptides, glycosphingolipids, Globo H, Lewis Y

## Abstract

Cancer stem cells (CSCs) are currently believed to be involved in tumor metastasis and relapse. And treatments against CSCs are well concerned issues. Peptides targeting to mouse and human CSCs were screened from an M13 phage display library. The first subset of cancer stem cell homing peptides (CSC HPs), CSC HP-1 to -12, were screened with mouse EMT6 breast cancer stem cells. Among them, CSC HP-1, CSC HP-3, CSC HP-8, CSC HP-9, and CSC HP-10 can bind to mouse CT26 colon CSCs; CSC HP-1, CSC HP-2, CSC HP-3, and CSC HP-8 can bind to mouse Hepa1-6 liver CSCs; as well as CSC HP-1, CSC HP-2, CSC HP-3, CSC HP-8, CSC HP-9, CSC HP-10, and CSC HP-11 can bind to human PANC-1 pancreatic CSCs. The second subset of cancer stem cell homing peptides, CSC HP-hP1 to -hP3, were screened with human PANC-1 pancreatic CSCs. Both CSC HP-hP1 and CSC HP-hP2 were demonstrated able to bind mouse EMT6, CT26 and Hepa1-6 CSCs as well as human colorectal HT29 and lung H1650 CSCs. CSC HP-1 and CSC HP-hP1 could strongly associate with the Globo 4 and Lewis Y glycan epitopes coupled on a microarray chip or Globo 4 and Globo H conjugated on bovine serum albumin. CSC HP-10, CSC HP-11 and CSC HP-hP2 could associate with the disialylated saccharide Neu5Ac-α-2,6-Gal-β-1,3-(Neu5Ac-α-2,6)-GalNAc coupled on a microarray chip. These results indicate that the CSC HPs may target to the known stem cell glycan markers GbH and Lewis Y as well as the disialylated saccharide.

## INTRODUCTION

Cancer stem cells (CSCs), also termed as tumor initiating cells (TICs), are operationally defined by their exclusive ability to initiate and maintain tumor growth and are small subpopulations of neoplastic cells within a tumor. Since the CD34^+^CD38^-^ fraction of human acute myeloid leukemia cells were found can disseminate in the severe combined immune-deficient (SCID) mice [1], TICs were soon identified in breast [2], brain [3], liver [4], colon [5], lung [6], and pancreatic tumors [7]. CSC is quiescent and divides asymmetrically to produce a dormant CSC and a highly proliferative progenitor cell. The asymmetry of CSC division is regulated by NOTCH signaling [8]. And the progenitor cells and their progenies compose the major mass of a tumor [9, 10]. Because CSCs are relatively quiescent within tumors, they are much less sensitive to anti-cancer drugs acting on highly proliferative cells and participated in tumor propagation after chemotherapies [11]. Additionally, CSCs have a high DNA repairing ability [12] and express high aldehyde dehydrogenase activity [13], ATP-binding cassette (ABC) transporter family proteins [14, 15], as well as anti-apoptosis proteins Bcl2 and Bclxl [for review, see 16-18]. CSCs prefer glycolysis rather than oxidative phosphorylation in energy metabolism [19, 20] and express a high level of hypoxia induced factor 1α (HIF1α) so as to sustain themselves in a low-oxygen environment [21]. According to these specificities, it is currently believed that CSCs are involved in tumor metastasis and relapse. And therapies aimed at CSCs are deeply concerned issues [22, 23].

CSCs can be enriched from tumor cells using various markers [10]. For example, CD44^+^, CD24^-^, and ALDH1^+^ were selected for breast CSCs [2, 24]. As few as 100 isolated cells were capable to colonize a tumor in SCID mice [2]. CD44^+^, CD133^+^, CD166^+^, CD24^+^, EpCAM^+^, ESA^+^, and ALDH1^+^ were set for colon CSCs [25–27]. CD133^+^ and EpCAM^+^ were focused for liver CSCs [28, 29]; as well as CD44^+^, CD133^+^, CD24^+^, ESA^+^, and ALDH1^+^ were picked for pancreas CSCs [30, 31]. The expression levels of the CSC markers on the cultured cancer cells are quite dynamic. The population of CD13^+^ cells in Li-7 hepatocellular carcinoma line decreased when the passage number increased [32].

Although epithelial-mesenchymal transition (EMT) is a key program mainly involved in cancer invasion and metastasis, it is also involved in generating the properties of stem cells. Ectopic expression of the EMT inducing factors, Twist or Snail, in transformed human mammary epithelial cells induced CD44^+^, CD24^-^ breast CSC phenotype [33]. Overexpression of Snail in human colorectal cancer cell lines, HT29 and HCT116 also increased the expression of CD44 and CD133 antigens [34]. Such kinds of cells cultured in two-dimensional (2-D) state cannot mimic CSCs precisely. Three-dimensional (3-D) cultures were developed to selectively expand normal and cancer mammary stem cells [35]. When the trypsinized single cell suspensions were cultured in serum-free media in ultra-low attachment plates at low density, the differentiated cells died because of anoikis [36, 37] and only stem cells could survive. Each stem cell could proliferate to a clump of cells named as mammosphere. As few as 500 cells of mammospheres could colonize a tumor on SCID mouse, therefore, the mammospheric cells were generally recognized as breast CSCs [35, 38, 39]. Serum-free 3-D cultures of other cancers, such as melanoma [40], brain tumor [3, 41], colon cancer [42], liver cancer [28], and pancreatic cancer [43] were also established and the cells composing of these cell clumps are termed as CSCs. Weiswald *et al.* suggested to nomenclature these cancer cell clumps prepare in serum-free media in non-attachment plates as tumorospheres in order to distinguish between multicellular tumor spheroids (MTSs) which were prepared in the presence of serum [44]. Under the last condition, the tumor cells of different types and differentiated states in a single cell suspension will aggregate to form organized and compact cell clumps [45, 46].

Although antibodies are currently used for cancer targeting, the tumor homing peptides (THPs) are superior in some aspects because of their small sizes. THPs are less immunogenic, deeper tumor penetrating and can be synthesized multi-valently and economically. Phage displayed random peptide libraries have been used and two general strategies are applied to screen THPs. In the first strategy, target proteins coated on the bottom of ELISA plates or cancer cells on culture plates were treated with phage libraries which were either pre-absorbed with non-specific proteins or normal cells. The phages absorbed on the targets were stripped and then amplified in host bacteria to get round-one enriched library. The panning procedures are usually repeated for three to seven rounds. By this strategy, the THPs specifically binding to either tumor antigens, such as HER2 [47] and CD21 [48], or to cancer cells, such as glioma [49], hepatoma [50], nasopharengeal carcinoma [51], and lung cancer cells [52-54], were reported. On the other hand, *in vivo* biopanning strategy was taken. Phage libraries were injected from the tail vein of a SCID mouse xenotransplanted with tumors. The phages entrapped inside the tumors were eluted and amplified as described above. The neoplastic vasculature is distinct from the normal one on the basis of structure and gene expression [55, 56]. It can be conceived that the majority of the THPs screened by the *in vivo* biopanning were verified associating with the endothelial cells of the neoplastic vasculatures. These THPs can be categorized as (i) peptides with RGD motif targeting to integrin α_v_β_3_ and α_v_β_5_ on the endothelia of the neoplastic blood vasculatures [57, 58], (ii) peptides with NGR motif targeting to CD13/aminopeptidase N on the endothelia of the neoplastic blood vasculatures [59, 60], and (iii) peptides targeting to neoplastic lymphatic vessels [61–63]. The THP sequence database, TumorHoPe, systematically collected from the published papers, patents and websites was released on-line [64].

In this report, CSCs prepared by the 3-D tumorosphere with serum-free culture methods from mouse breast, colon, and liver cancer cell lines as well as human pancreatic cancer cell line were used to screen peptides which prefer binding to CSCs than cancer cells (CCs). A subset of cancer stem cell homing peptides (CSC HPs) was selected from a phage displayed random peptide library by mouse breast CSCs. These CSC HPs can recognize mouse colon and liver CSCs as well as human pancreatic CSCs, even with various tendencies. Another subset of CSC HPs screened by human pancreatic CSCs was collected too. They can recognize mouse CSCs vice a versa. The targets of these CSC HPs have been explored and some possible candidates are reported hereafter.

## RESULTS

### Surface markers of CSCs

**Figure 1:**
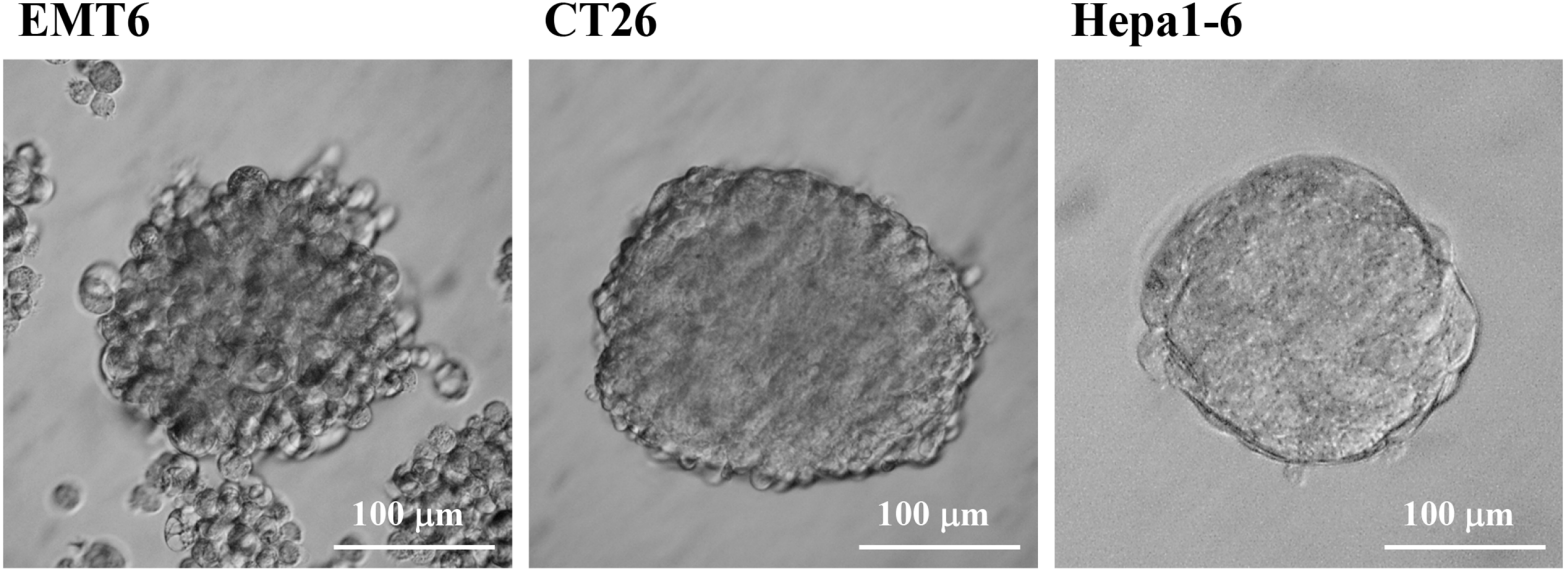

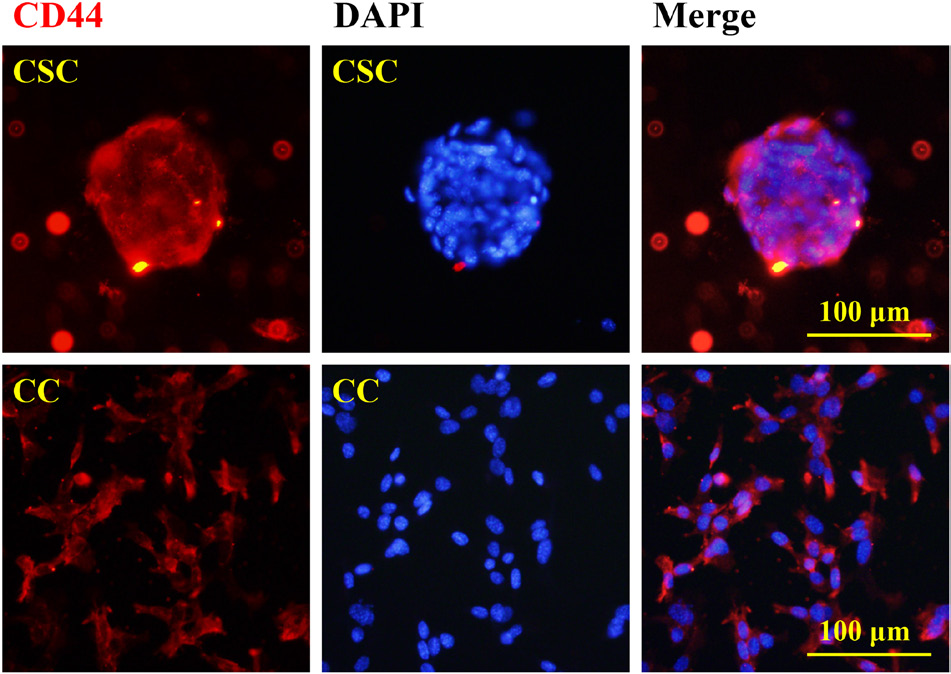

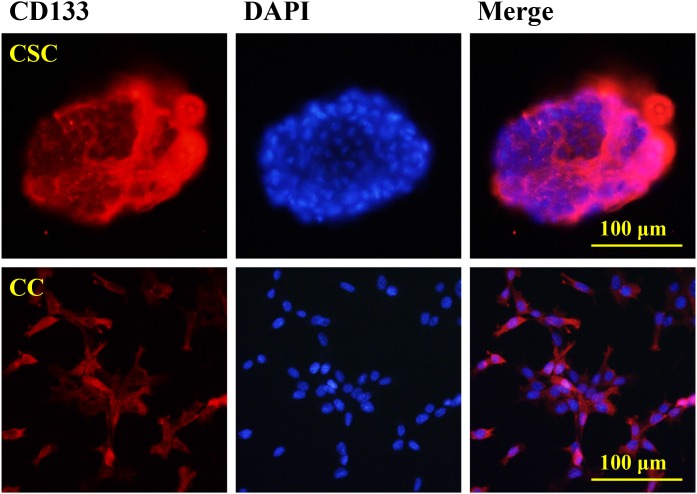

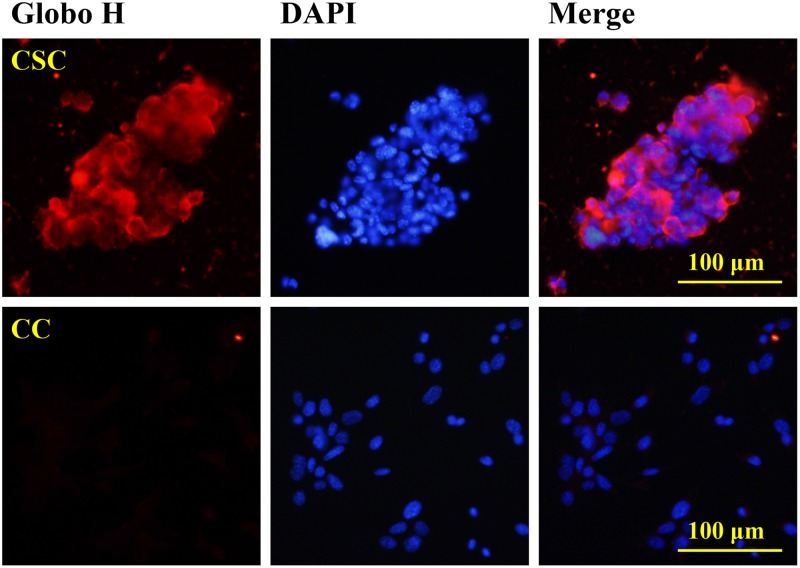

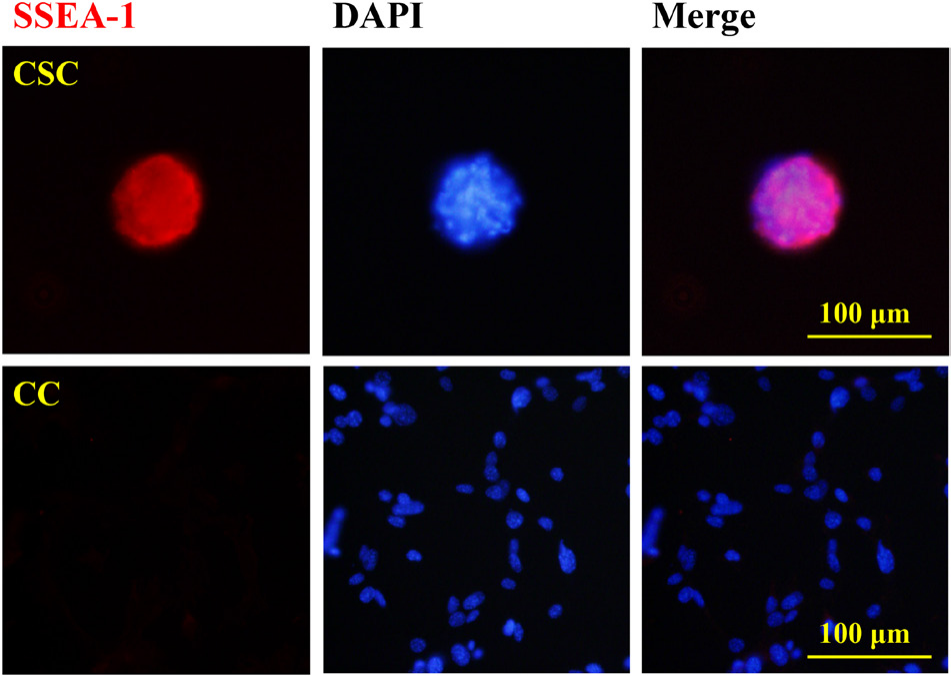

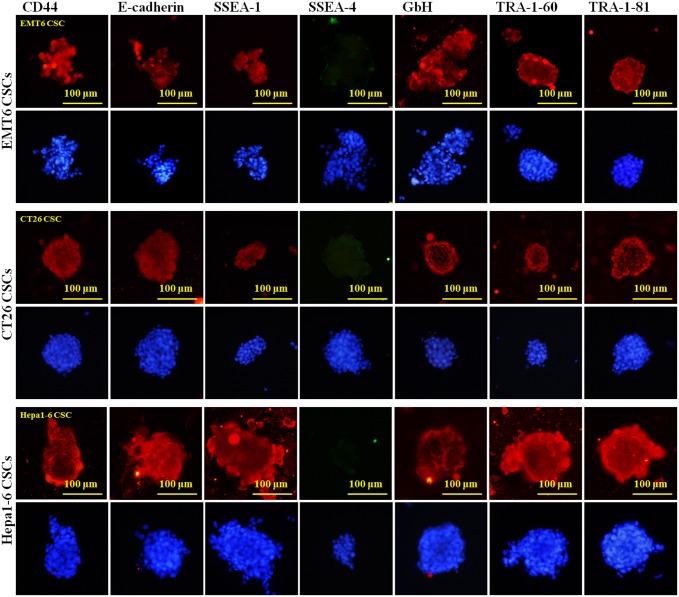
Preparation and characterization of mouse CSCs **(A)** Tumorospheres of mouse EMT6 breast cancer cells (left panel), CT26 colon cancer cells (middle panel), and Hepa1-6 liver cancer cells were prepared. **(B)** EMT6 CSCs and CCs were incubated with primary antibodies against CD44, CD133, Globo H (GbH), and SSEA-1 epitopes on EMT6 CSCs and CCs. Either DyLight594-conjugated goat anti-Rabbit IgG for CD44 and CD133 or DyLight594-conjugated goat anti-mouse IgM for Globo H and SSEA-1 were used as secondary antibodies. **(C)** The IF images of EMT6, CT26 and Hepa1-6 CSCs were performed with primary antibodies against CD44, E-cadherin, SSEA-1, SSEA-4, GbH, TRA-1-60, and TRA-1-81 antibodies followed with secondary antibodies DyLight594-conjugated goat anti-Rabbit IgG for CD44 and E-cadherin, DyLight594-conjugated goat anti-mouse IgM for SSEA-1, GbH, TRA-1-60, and TRA-1-81, as well as DyLight488-conjugated donkey anti-mouse IgG for SSEA-4. The scaling bars of 100 μm are shown.

Although CD44 and CD133 have been used to enrich CSCs from human breast cancer [2, 24], colon cancer [25–27], and liver cancer [28, 29], CD44 was detected on the mouse breast EMT6, colon CT26, and liver Hepa1-6 CCs. CD133 is also monitored on EMT6 CCs (Figure 1B). Another set of CSC surface markers were needed to distinguish these CSCs from CCs. Since stage-specific embryonic antigen-3 (SSEA-3) has been reported specific for human breast CSCs [65], a series of antibodies against early embryonic developmental markers [66, 67] were tested. Like human cancer cell lines, mouse CSCs can be prepared as tumorospheres by the serum-free culture methods (Figure 1A). Besides SSEA-1 and GbH which were detected on EMT6 CSCs rather than CCs (Figure 1B), the embryonic stem cell (ESC) markers, SSEA-1, GbH, TRA-1-60 and TRA-1-81, as well as epithelial marker E-cadherin were just detected on the CSCs of EMT6, CT26 and Hepa-1 (Figure 1C) but not on the CCs. Like mouse ESCs, SSEA-4 was not detected on mouse CSCs.

### Screening of CSC specific HPs from a phage display library

**Figure 2:**
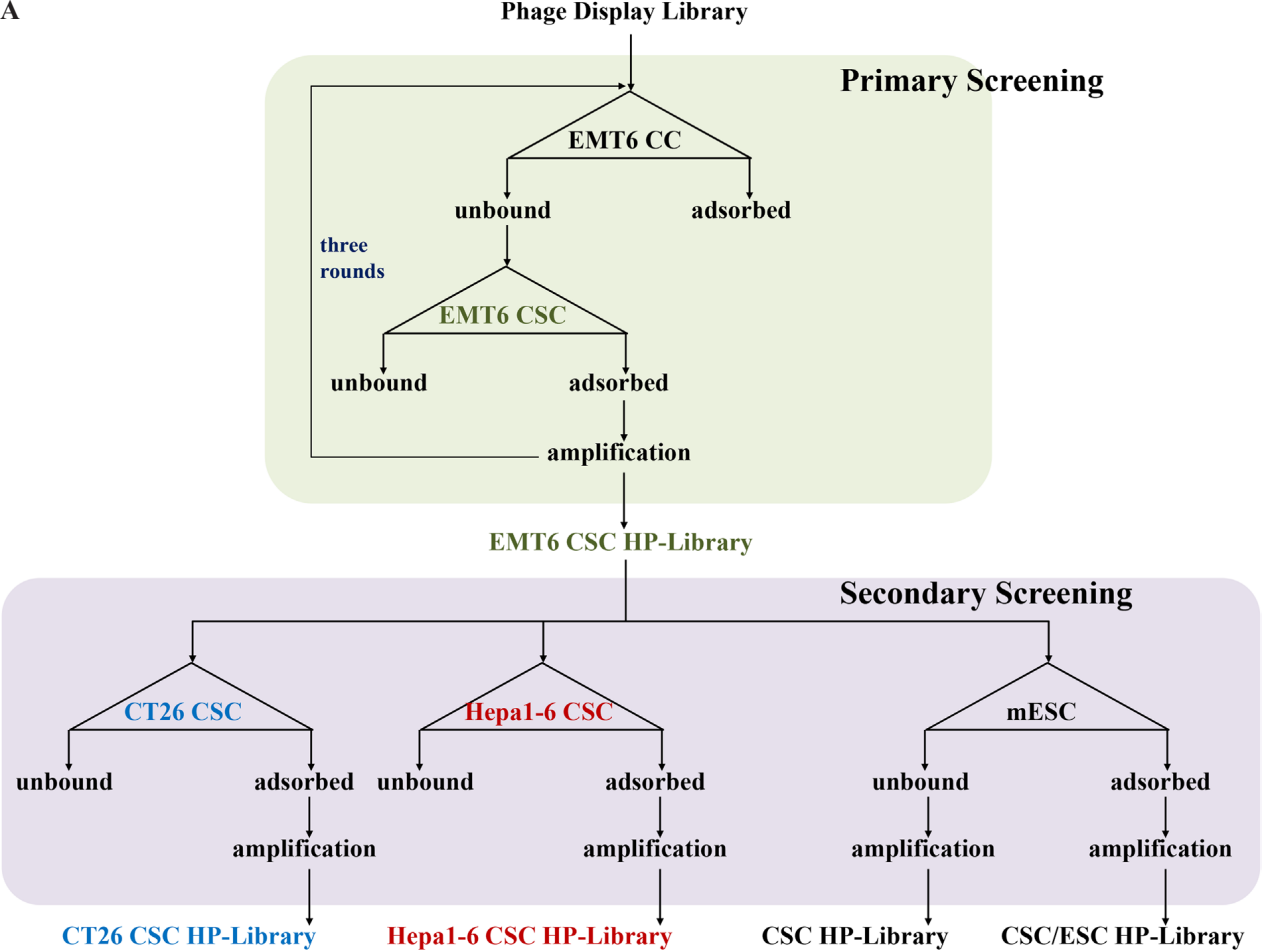

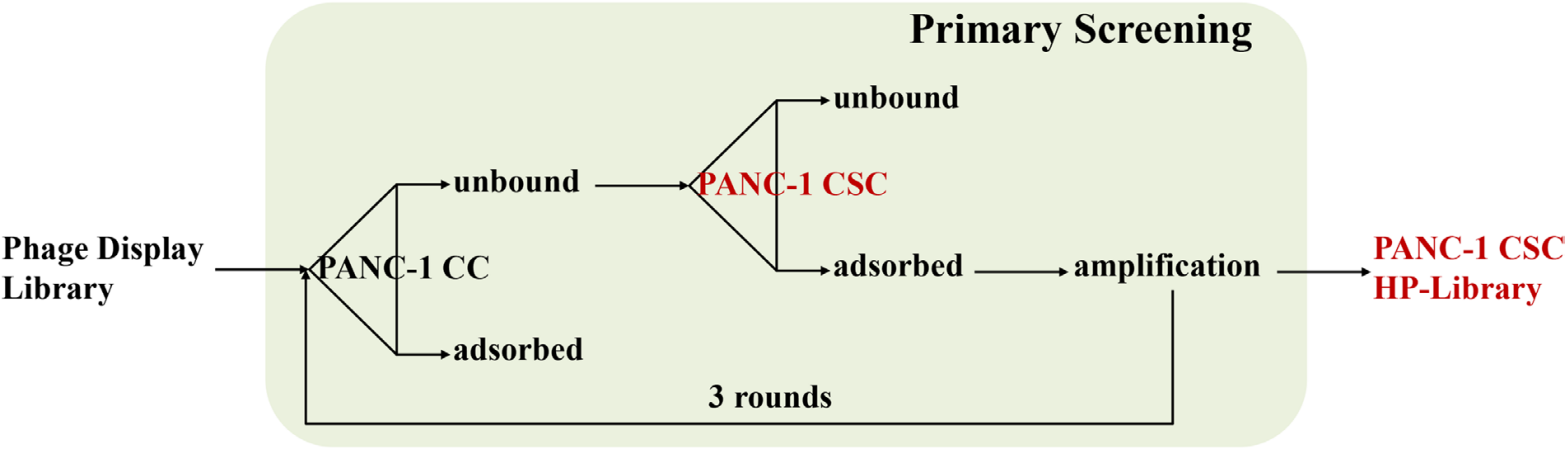
Strategies for the establishment of primary and secondary CSC HP libraries **(A)** Flow chart of the primary screening from the original M13 PhD7 library to prepare the EMT6 CSC HP-Library was shown in the upper part. The secondary screening tactics to produce the CT26 CSC HP-Library, the Hepa1-6 CSC HP-Library, the CSC/ESC HP-Library, and the CSC HP-Library was illustrated in the lower part. **(B)** The same strategy of EMT6 CSC HP-library preparation was used to establish the PANC-1 CSC HP-library.

The flow chart of strategy to explore mouse CSC specific HPs is shown in Figure 2A. The M13 PhD7 hepta-peptide library was pre-adsorbed by EMT6 CCs to remove the general cancer HPs, then the free phages were transferred to EMT6 CSCs for positive selection. The phages associated on EMT6 CSCs were trapped and amplified by *E. coli* ER2738. The aforementioned selection procedures were carried out for three rounds to prepare an enriched primary EMT6 CSC HP library. Independent plaques were randomly picked and 31 valid sequences were read and summarized in Table 1. Peptide SQPTWMF (signed as CSC HP-1), GMMSSPP (CSC HP-2), FSGGGNH (CSC HP-3), FPFTKNL (CSC HP-4), and ATYGNLW (CSC HP-5) occurred 10, 6, 5, 3, and 2 times, respectively. The other peptides, YHMPALM (CSC HP-6), HGGVRLY (CSC HP-7), ELTPLTL (CSC HP-8), GPSASRN (CSC HP-10), and GLAPFNA (CSC HP-11) appeared once. Since the M13 PhD7 library was estimated to contain 10^9^ independent clones, it can be concluded that the selection procedures were highly effective.

**Table 1:** The times of CSC HP sequences detected in the primary and secondary CSC HP-libraries

CSC HP		Primary	Secondary			
Name	Sequence	EMT6 (n=31)	CT26 (n=20)	Hepa1-6 (n=10)	CSC/ESC (n=20)	CSC (n=20)
CSCHP-1	SQPTWMF	**10**	6	0	**11**	8
CSCHP-2	GMMSSPP	**6**	3	0	1	1
CSCHP-3	FSGGGNH	5	1	**10**	1	1
CSCHP-4	FPFTKNL	3	0	0	2	2
CSCHP-5	ATYGNLW	2	1	0	1	2
CSCHP-6	YHMPALM	1	1	0	1	**2**
CSCHP-7	HGGVRLY	1	1	0	1	**2**
CSCHP-8	ELTPLTL	1	**7**	0	2	0
CSCHP-9	KIYTTLD	0	0	0	0	**1**
CSCHP-10	GPSASRN	**1**	0	0	0	0
CSCHP-11	GLAPFNA	**1**	0	0	0	0
CSCHP-12	NLQPPAY	0	0	0	0	**1**

The primary CSC HP-library was further selected directly once by either CT26 or Hepa1-6 CSCs to test the bias of CSC HP affinities to different kinds of cancers. As the data show in Table 1, CSC HP-8 and CSC HP-3 were more attractive for CT26 and Hepa1-6 CSCs, respectively. The bias of CSC HP affinities between CSCs and embryonic stem cells (ESCs) was another interesting issue. The CSC HP-1 was further enriched in the ESC bound fraction (CSC/ESC-HP-library). Two new peptide sequences, KIYTTLD (CSC HP-9) and NLQPPAY (CSC HP-12), were read in the ESC unbound fraction (CSC HP-library) (Table 1).

### Interactions between CSC HPs and CSCs

**Figure 3:**
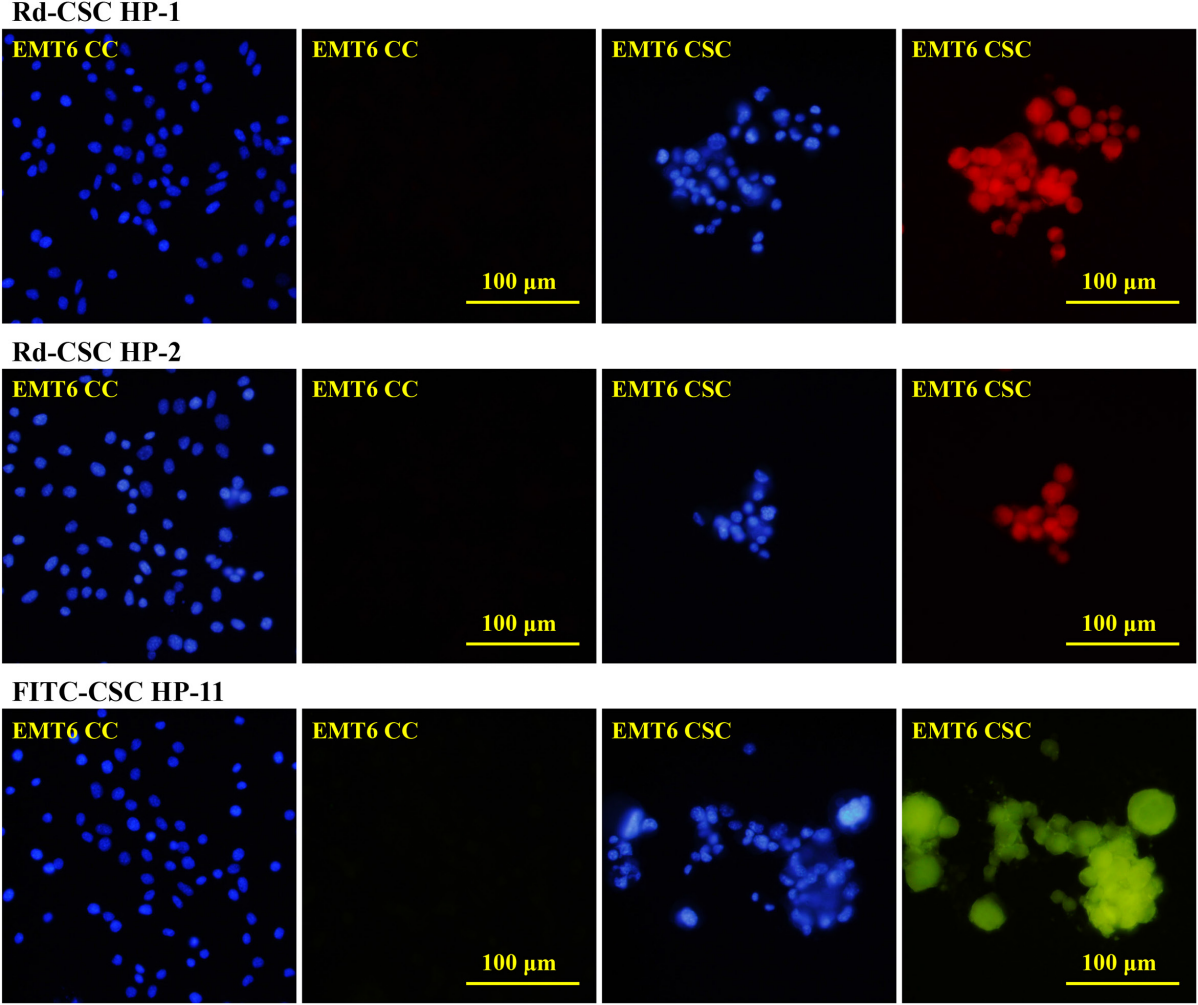

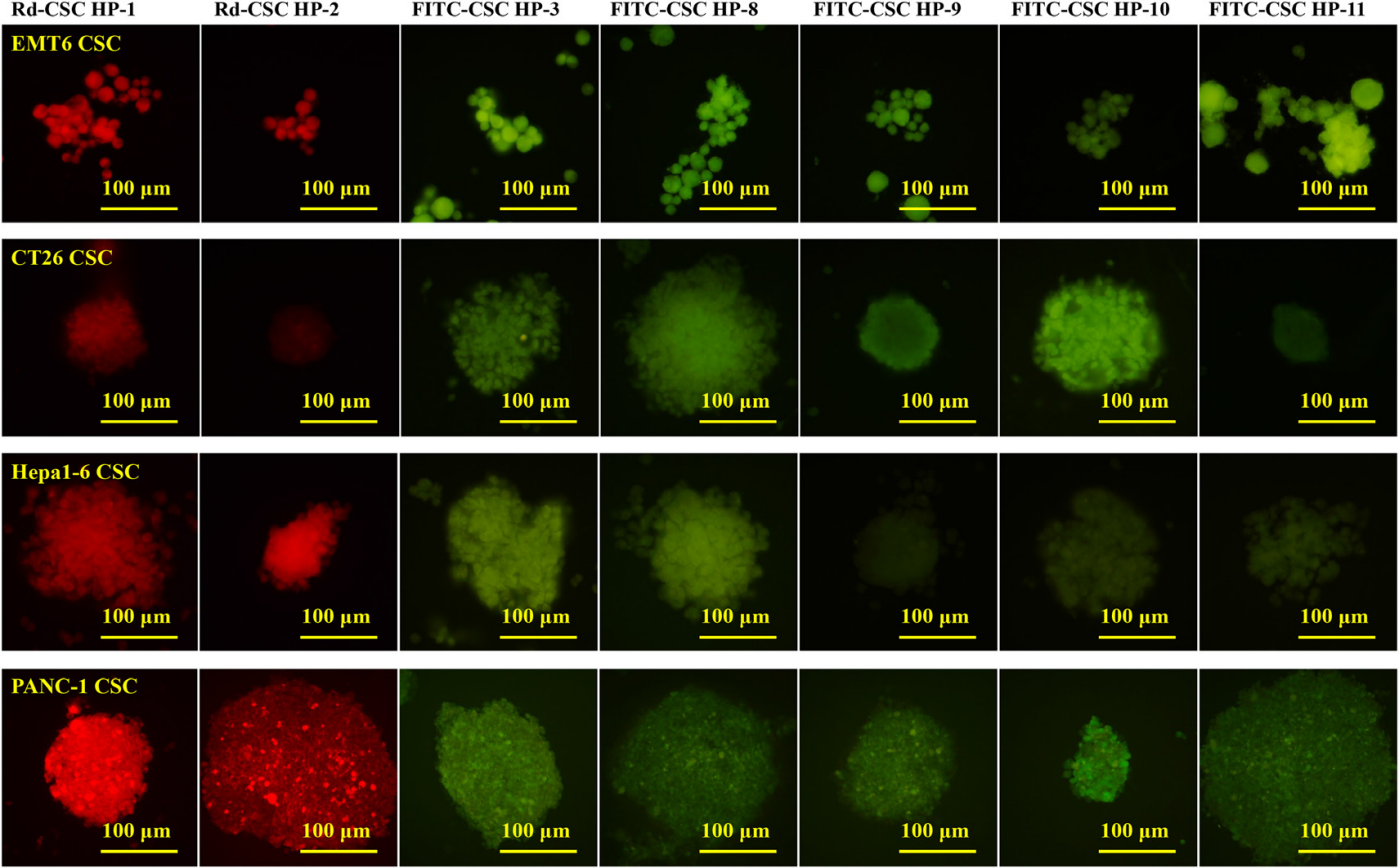
Selective binding of CSC HPs to CSCs **(A)** Rhodamine B labeled CSC HP1 (Rd-CSC HP-1) and CSC HP2 (Rd-CSC HP-2) as well as FITC labeled CSC HP11 (FITC-CSC HP-11) were directly used to stain EMT6 CSCs and CCs. Cells were counter-stained with DAPI. **(B)** The CSCs of EMT-6, CT26, Hepa1-6 and PANC-1 were separately stained with Rd-CSC HP-1, Rd-CSC HP-2, FITC-CSC HP-3, FITC-CSC HP-8, FITC-CSC HP-9, FITC-CSC HP-10 or FITC-CSC HP-11. Fluorescence images were captured with the same exposure conditions. Either 50 μg/mL of Rd-CSC HPs or FITC-CSC HPs were used to stain these CSCs. All the pictures were of the same magnification and taken with 40× objective lens. The scaling bars of 100 μm are shown on the right panel pictures.

According to these results, CSC HP-1, CSC HP-2, CSC HP-3, CSC HP-8, CSC HP-9, CSC HP-10, and CSC HP-11 were selected and chemically synthesized with N-terminal conjugation with rhodamine B (Rd-CSC HPs) or fluorescein (FITC-CSC HPs) as listed in Table 2. All of these peptides specifically associated with EMT6, CT26 and Hepa1-6 CSCs. They were not detectable in the parallel CC experiments (Figure 3A and 3B). It was of interest to determine whether these CSC HPs derived from mouse cancers could recognize human CSCs. Tumorospheres prepared from the human pancreatic cancer cell line PANC-1 were found to be associated with the seven peptides. (Figure 3B)

**Table 2:** Chemically synthesized Rhodamine-, Fluorescein- and Biotin-conjugated CSC HPs

Conjugation	Rhodamine B	Fluorescein	Biotin
CSC HP-1	Rd-GSQPTWMF		Biotin-GGSQPTWMF
CSC HP-2	Rd-GMMSSPP		Biotin-GGMMSSPP
CSC HP-3		FITC-GFSGGGNH	Biotin-GGFSGGGNH
CSC HP-4			Biotin-GGFPFTKNL
CSC HP-5			Biotin-GGATYGNLW
CSC HP-6			Biotin-GGYHMPALM
CSC HP-7			Biotin-GGHGGVRLY
CSC HP-8		FITC-GELTPLTL	Biotin-GGELTPLTL
CSC HP-9	Rd-GKIYTTLD	FITC-GKIYTTLD	Biotin-GGKIYTTLD
CSC HP-10		FITC-GPSASRN	Biotin-GGPSASRN
CSC HP-11		FITC-GLAPFNA	Biotin-GGLAPFNA
CSC HP-12			Biotin-GGNLQPPAY
CSC HP-hP1	Rd-GGPKVTIW		Biotin-GGPKVTIW
CSC HP-hP2	Rd-GGSPVMSW		Biotin-GGSPVMSW
CSC HP-hP3			Biotin-GGYHQVKPH

### Primary PANC-1 CSC HP Library

**Figure 4:**
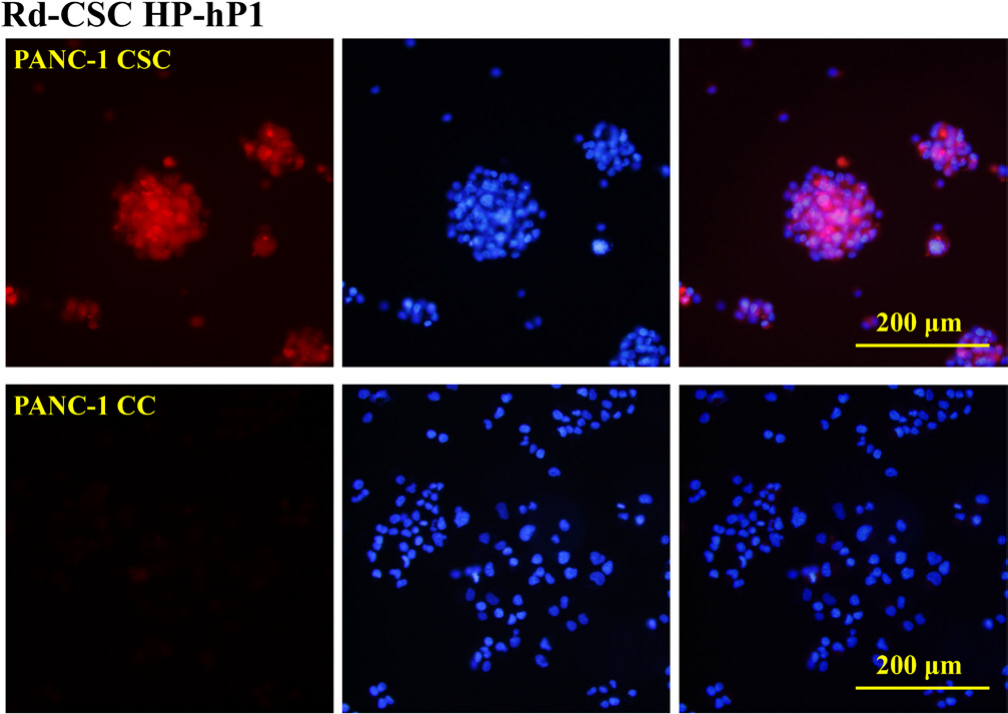

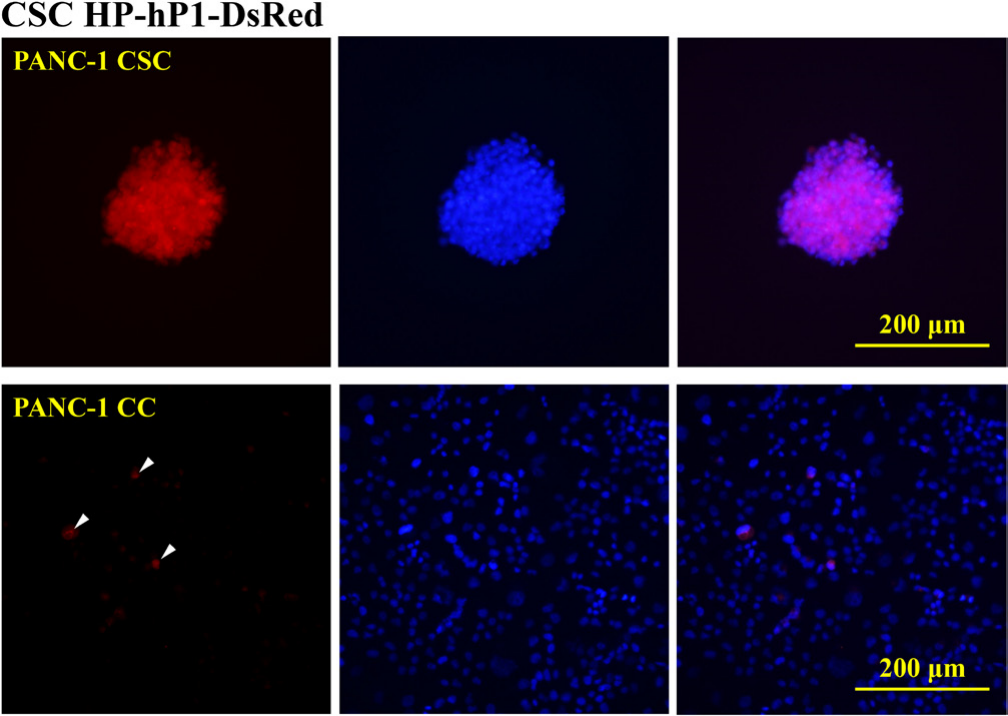

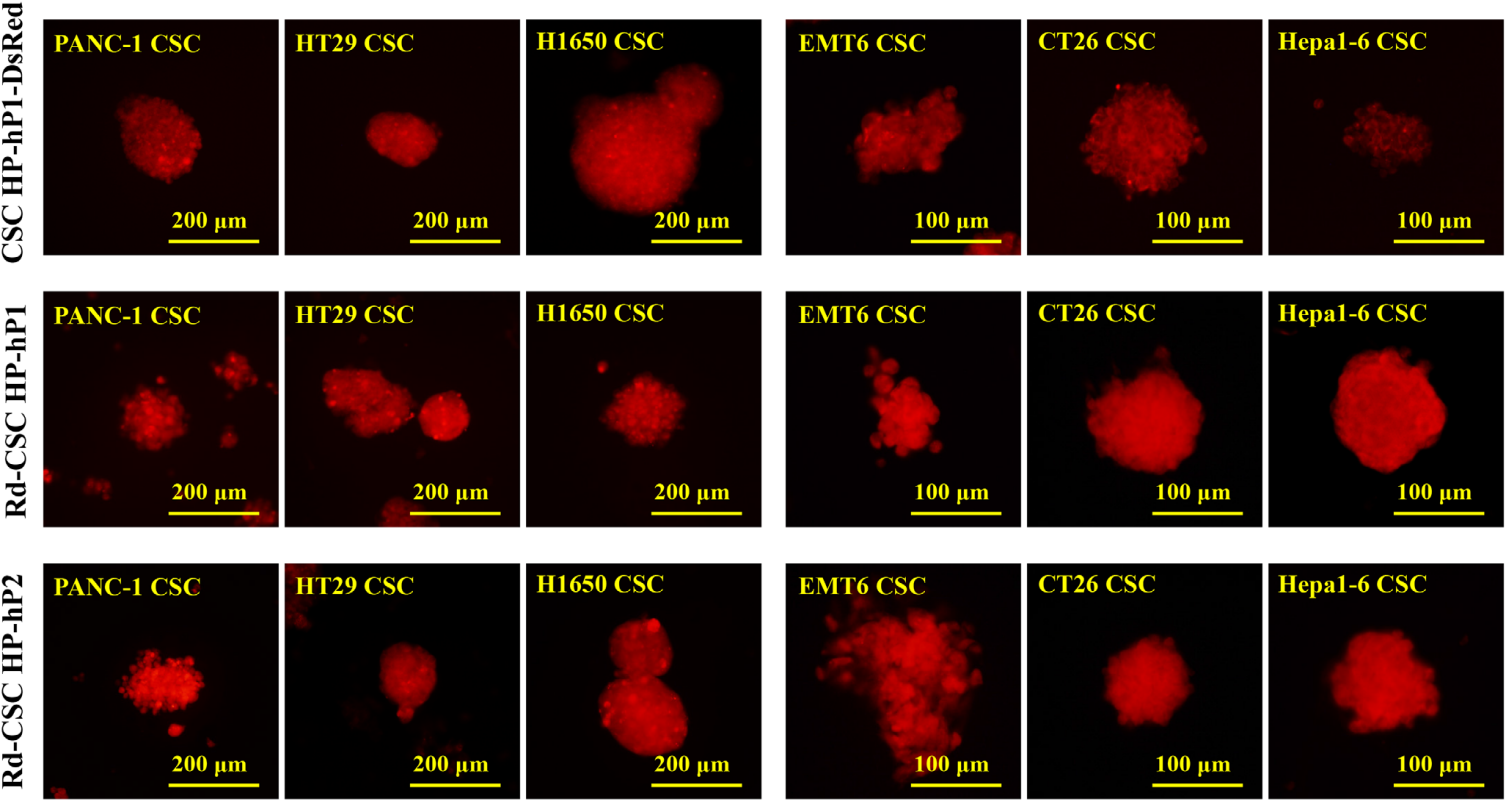
Selective binding of CSC HP-hPs to CSCs **(A)** PANC-1 CSCs and CCs were stained with 50 μg/mL of Rd-CSC HP-hP1. **(B)** PANC-1 CSCs and CCs were stained with 25 μg/mL of CSC HP-hP1-DsRed recombinant protein. Some PANC-1 CCs stained were indicated by arrowheads. **(C)** The CSCs of human PANC-1, HT29 and H1650 as well as mouse EMT6, CT26 and Hepa-1 were stained with 25 μg/mL of CSC HP-hP1-DsRed recombinant protein (upper panels, 50 μg/mL of Rd-CSC HP-hP1 (middle panels), or 50 μg/mL of Rd-CSC HP-hP2 (lower panels). The pictures of human and mouse CSCs were taken with 20× and 40× objective lens, respectively. The scaling bars of 200 μm for human CSCs or 100 μm for mouse CSCs are presented.

If the CSC HPs selected by mouse CSCs could cross-react with human CSCs, could the CSC HPs selected by human CSCs associate with mouse CSCs? Following the similar procedures of EMT6 CSC HP screening, after three rounds of negative selection with human pancreatic PANC-1 CCs and positive selection with PANC-1 CSCs, a primary PANC-1 CSC HP-library was established. Independent plaques were randomly picked and 20 valid sequences were read. Peptide GPKVTIW (as signed as CSC HP-hP1), GSPVMSW (CSC HP-hP2), and YHQVKPH (CSC HP-hP3) occurred 17, 2, and 1 times, respectively. (Table 3) The amino acid sequences of the CSC HP-hP series did not match those of the mouse CSC HPs. In addition to chemically synthesized Rd-CSC HP-hP1, which could selectively associate with human PANC-1CSCs (Figure 4A). Besides PANC-1 CSCs, Rd-CSC HP-hP1 and Rd-CSC HP-hP2 could also associate with human colorectal cancer HT29 and lung cancer H1650 CSCs as well as mouse EMT6, CT26 and Hepa-1CSCs (Figure 4C), CSC HP-hP1-DsRed recombinant protein was prepared to test whether the interactions between CSC HP-hP1 and CSCs would be affected by a large molecule cargo fused to the C-terminus of CSC HP-hP1 peptide. The CSC HP-hP1-DsRed recombinant protein was demonstrated able to bind to all of the six kinds of CSCs tested (Figure 4B and 4C).

**Table 3:** The times of CSC HP sequences detected in the primary PANC-1CSC HP-libraries

CSC HP		Primary
Name	Sequence	PANC-1 (n=20)
CSC HP-hP1	GPKVTIW	17
CSC HP-hP2	GSPVMSW	2
CSC HP-hP3	YHQVKPH	1

### Targets of the CSC HPs

**Figure 5:**
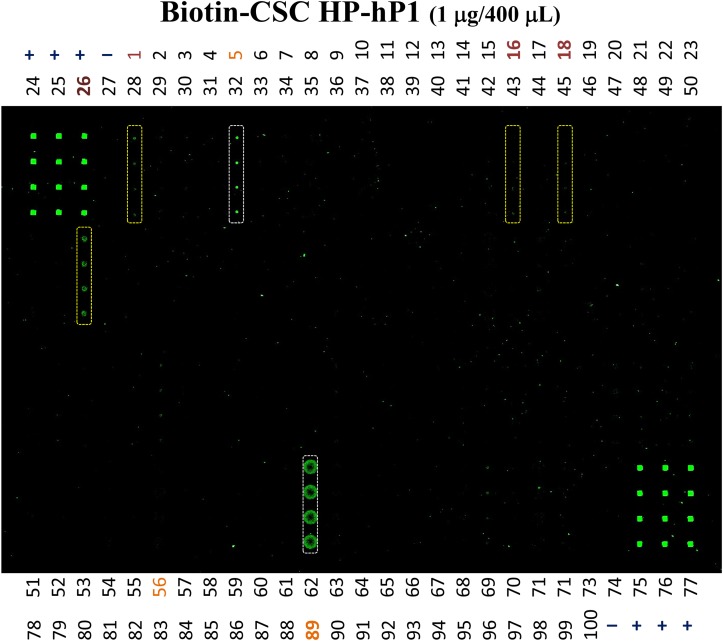

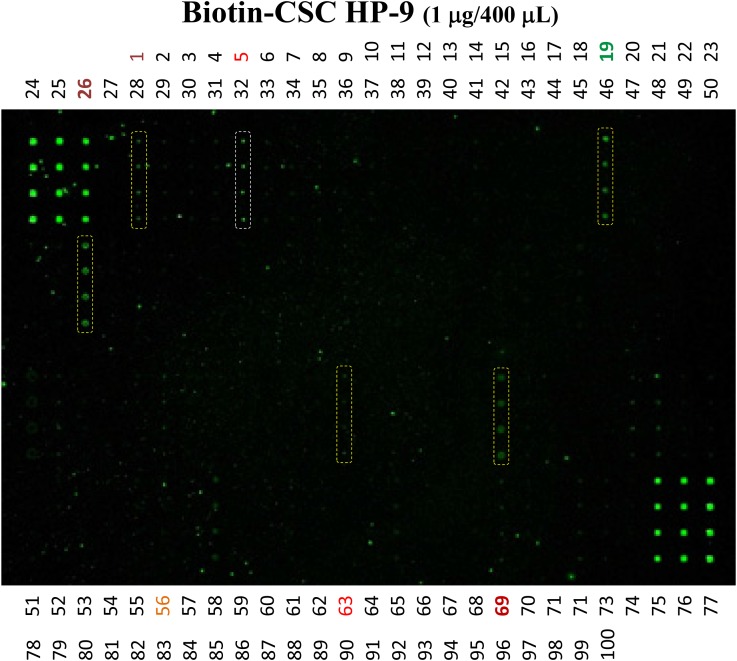

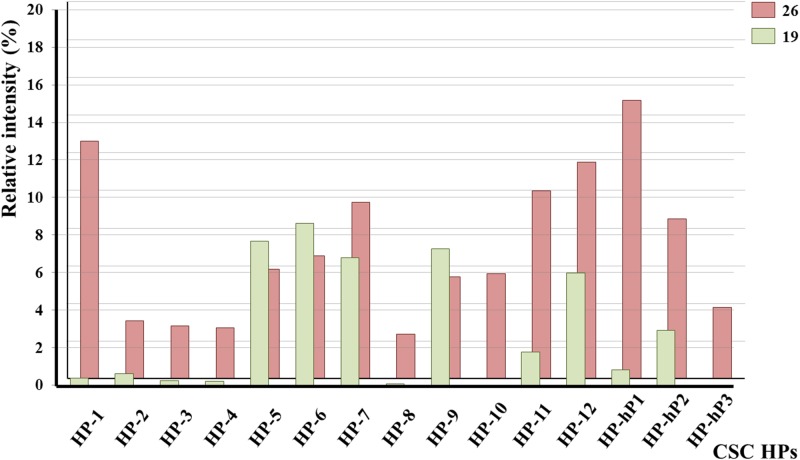

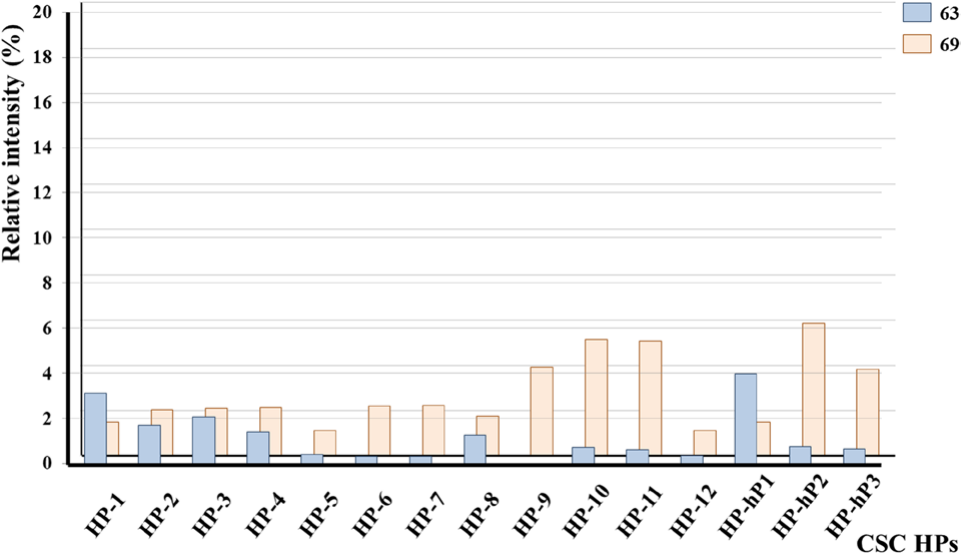

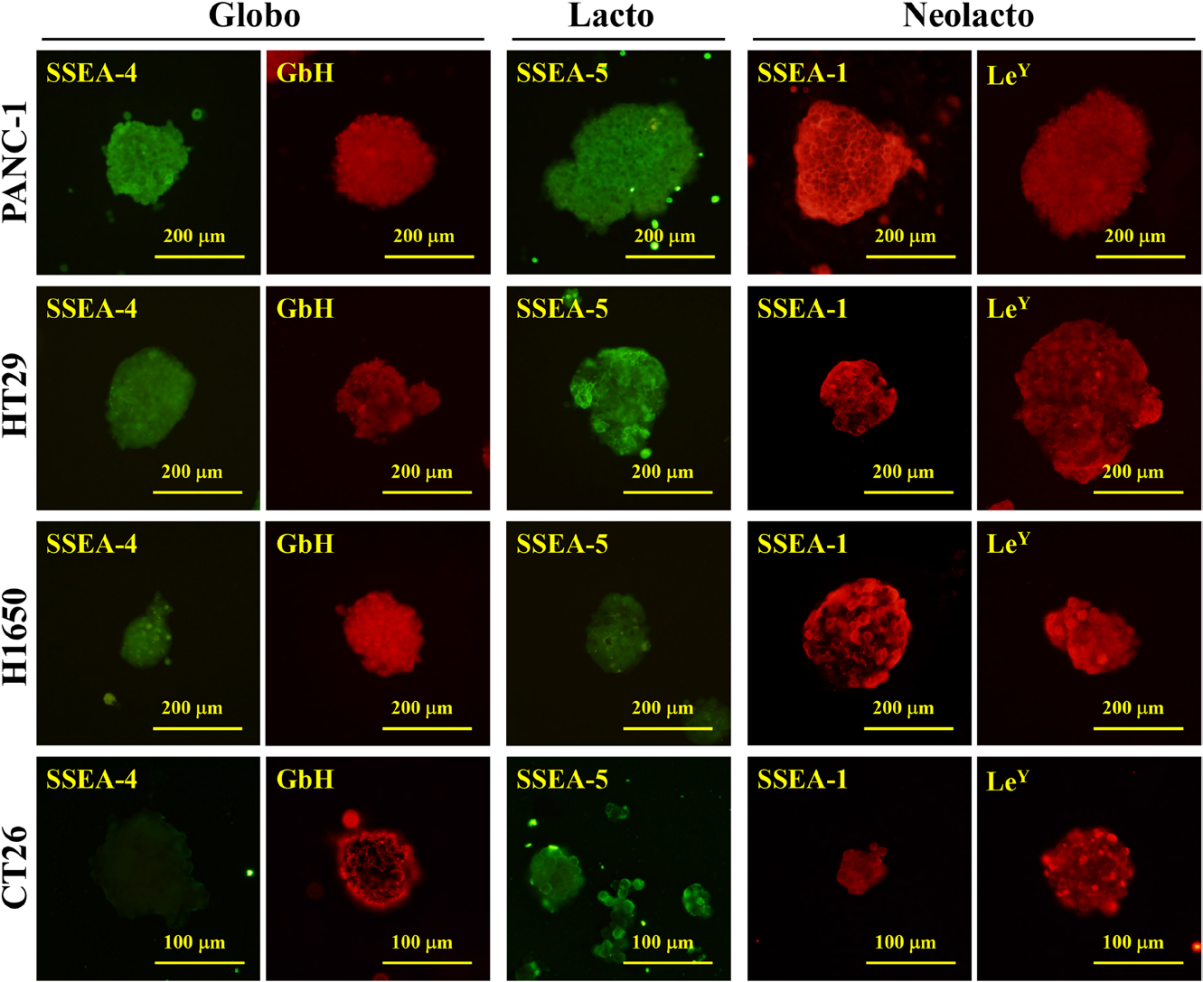
Targets of CSC HPs analyzed by glycan microarray Glycan microarray analysis of CSC HP-hP1 **(A)** and CSC HP-9 **(B)** labeled with biotin was performed. The tetra-plicate spots of interest were marked with a yellow dash line. Strong signals, such as glycan 5 (α-Rha) and glycan 89 (β-1,4-Xylotetraose), without apparent significance were marked with a white dash line. **(C)** The average relative intensities of Globo (glycan 26) versus Lacto/Neolacto (glycan 19) signals, which were normalized with the positive control signal 3 of the same micro array collected from the 15 CSC HPs, were plotted. The maximal standard derivation for glycan 19 and 26 are 0.033% and 0.019%, respectively. **(D)** The relative intensities of glycan 63 and glycan 69 signals from the 15 CSC HPs were plotted. The maximal standard derivation for glycan 63 and 69 are 0.012% and 0.007%, respectively. **(E)** The IF images of stem-cell-specific glycan markers on human PANC-1, HT29, H1650, and mouse CT26 CSCs were performed with monoclonal antibodies against Globo series (SSEA-4 and GbH), Lacto series (SSEA-5), and Neolacto series (SSEA-1 and Le^Y^).

The CSC HPs isolated could distinguish CSCs from CCs, and this phenomenon was found cross-species between human and mouse. It was very interesting to figure out the targets of these CSC HPs. There were no detectable fluorescent signals of FITC-CSC HPs remaining on the nitrocellulose membrane that had been blotted with CSC membrane proteins separated by SDS PAGE (data not shown). Glycan microarray (Glycan Array 100 microchips), on which 100 kinds of oligosaccharides were spotted 4 times, was utilized to analyze the possibilities of the interactions between oligosaccharides and CSC HPs. Biotin-CSC HPs as listed in Table 2 were incubated with glycans on the chips. The biotin-CSC HPs trapped on the chips were explored with Cy3-conjugated streptavidin, and the fluorescent signals on them were read by a laser scanner. Two major patterns were illustrated in Figure 5. The first one included glycan 16 (Gal-β-1,4-Glc-β-), 18 (Gal-α-1,4-Gal-β-1,4-Glc-β-, also called as Gb3), and 26 (GalNAc-β-1,3-Gal-α-1,4-Gal-β-1,4-Glc-β-, also called as Gb4) Globo series (Figure 5A). Typical cases were CSC HP-1 and CHC HP-hP1 (Figure 5C). In addition to the Globo series signals, the second major pattern involved the glycan 19 (GlcNAc-β-1,3-Gal-β-1,4-Glc-β-, the Lacto tri-saccharide Lc3), which was similar to the level of signal 26 (Figure 5B). The typical cases were CSC HP5, CSC HP6, CSC HP7, and CSC HP9 (Figure 5C). Another two glycan markers, signal 63 (Fuc-α-1,2-Gal-β-1,4-(Fuc-α-1,3)-GlcNAc-β-, also called Lewis Y (Le^Y^) epitope) and signal 69 (Neu5Ac-α-2,6-Gal-β-1,3-(Neu5Ac-α-2,6)-GalNAc-β-), were also detected. CSC HP-1 and CSC HP-hP1 performed higher signals on glycan 63, and CSC HP-9, CSC HP-10, CSC HP-11, CSC HP-hP2, and CSC HP-hP3 performed higher signals on glycan 69 (Figure 5D). According to these results, the stem cell markers of the Globo series, SSEA-4 and GbH, of the Lacto series, SSEA-5 and of the Neolacto series, SSEA-1 and Le^Y^, on the CSCs of human PANC-1 and HT29 as well as mouse CT26 were analyzed by IF assays. Except SSEA-4 was not found on CT26 CSCs, all of the five glycan antigens were detected on these CSCs (Figure 5E).

**Figure 6:**
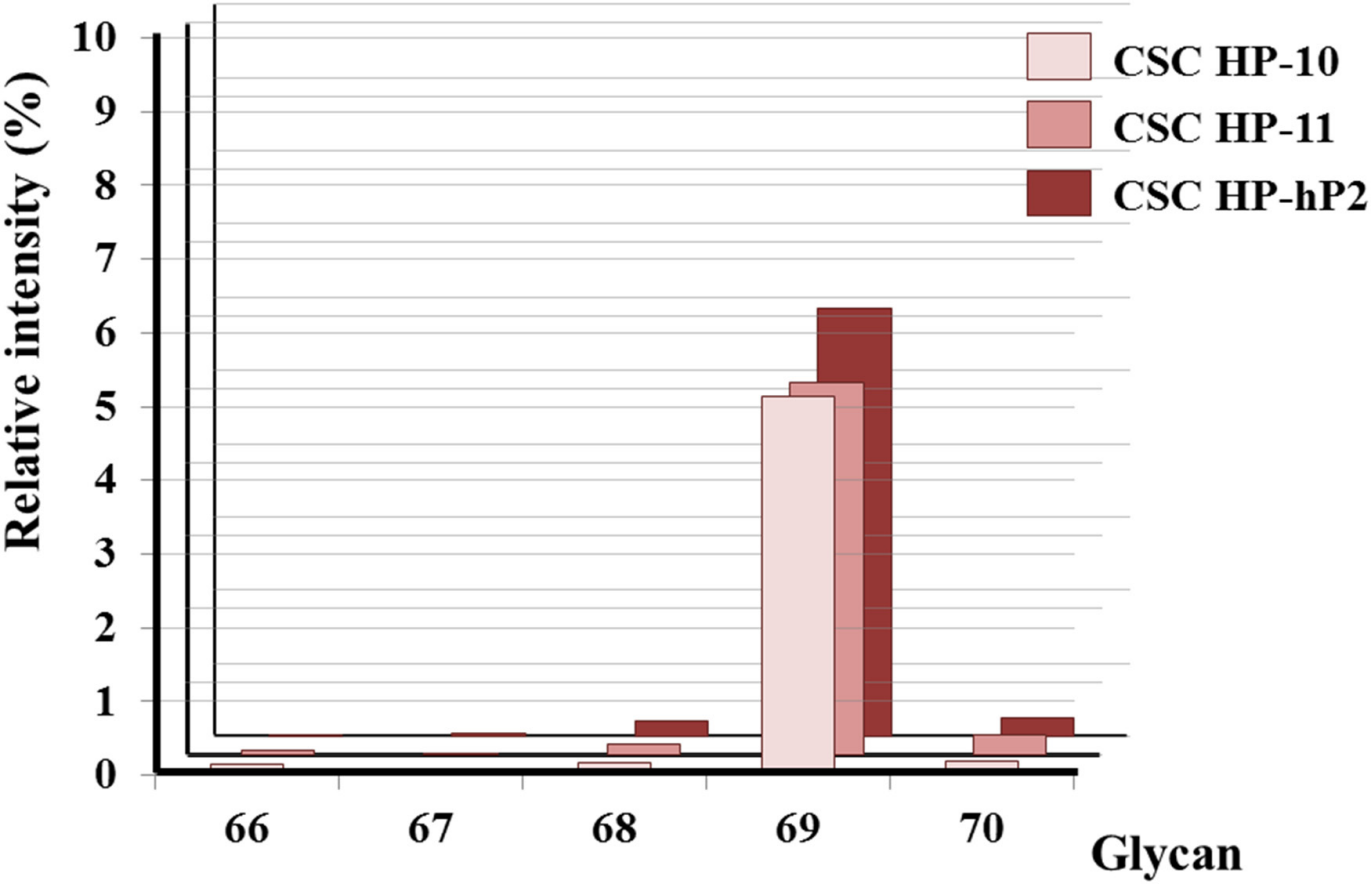

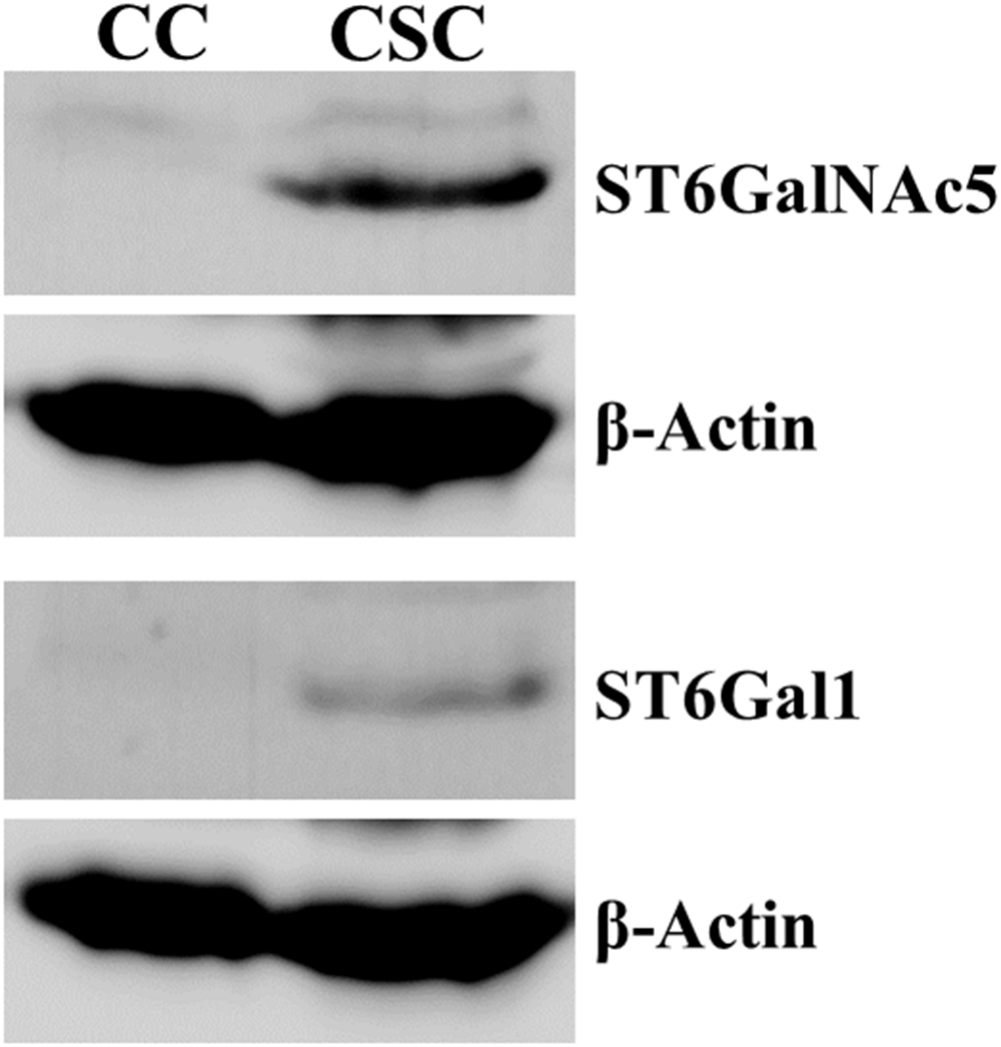
Analysis of α-2,6-sialylation on the terminal Gal-β-1,3-GalNAc- glycan moiety **(A)** The relative intensities of glycan 66 (Gal-β-1,3-GalNAc-β-), glycan 67 (Gal-β-1,3-(Neu5Ac-α-2,6)-GalNAc-β-), glycan 68 (Neu5Ac-α-2,6-Gal-β-1,3-GalNAc-β-), glycan 69 (Neu5Ac-α-2,6-Gal-β-1,3-(Neu5Ac-α-2,6)-GalNAc-β-), and glycan 70 (Neu5Ac-α-2,3-Gal-β-1,3-(Neu5Ac-α-2,6)-GalNAc-β-) signals collected from CSC HP-10, CSC HP-11, and CSC HP-hP2 chips were plotted. **(B)** Western blotting analysis of ST6GalNAc5 and ST6Gal1 protein expression in PANC-1 CCs and CSCs. β-actin was illustrated as internal control.

The dependence on the geometry of the two sialic acid linked to the terminal Gal-β-1,3-GalNAc- glycan was studied by analyzing the relative signal intensities of glycan 66 (Gal-β-1,3-GalNAc-β-), glycan 67 (Gal-β-1,3-(Neu5Ac-α-2,6)-GalNAc-β-), glycan 68 (Neu5Ac-α-2,6-Gal-β-1,3-GalNAc-β-), glycan 69 (Neu5Ac-α-2,6-Gal-β-1,3-(Neu5Ac-α-2,6)-GalNAc-β-), and glycan 70 (Neu5Ac-α-2,3-Gal-β-1,3-(Neu5Ac-α-2,6)-GalNAc-β-) (Supplementary Figure 3). Both of the two Neu5Ac-α-2,6- linkages are necessary for the associations with the CSC HPs (Figure 6A). The enzymes, ST6GalNAc5 and ST6Gal1, involved in the formation of these two likages were analyzed by western blotting method. Both of the two proteins were selectively expressed in PANC-1 CSCs (Figure 6B).

### The interactions between CSC HPs and Gb4 and GbH

**Figure 7:**
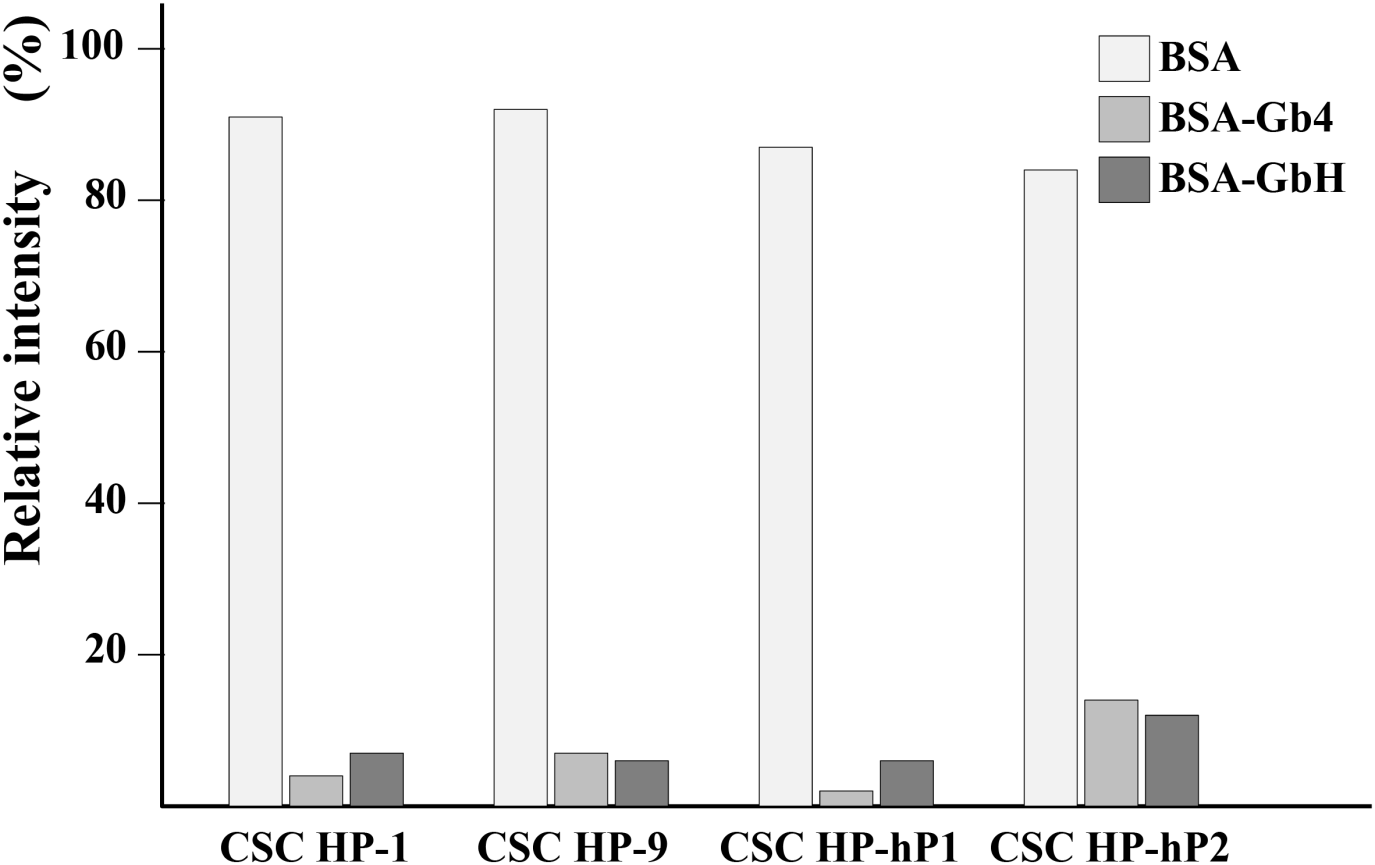
Binding assay of CSC HPs on Gb glycans conjugated on BSA The rodomine labeled CSC HPs, Rd-CSC HP-1, Rd-CSC HP-9, Rd-CSC HP-hP1, and Rd-CSC HP-hP2, were mixed with equimolar of BSA, Gb4-BSA or GbH-BSA. The free form of these Rd-CSC HPs were separated from which associated with BSA or Gb-BSA by ultra-filtration. In the absence of BSA or Gb-BSA, the relative fluorescence intensity of these Rd-CSC HPs in the filtrate were defined as 100%.

The longest globo series glycan spotted on the Glycan Array 100 is Gb4, however, Gb4 is an intermediate during the stem cell antigens SSEA-4 and GbH synthesis pathway (Supplementary Figure 2). The interactions between CSC HPs and Gb4 or GbH were monitored by the free form of rhodamine conjugated CSC HPs in the presence of either Gb4 or GbH chemically coupled to BSA (Gb4-BSA and GbH-BSA, respectively). As data shown in Figure 7, Rd-CSC HP-1, Rd-CSC HP-9, Rd-CSC HP-hP1, and Rd-CSC HP-hP2 were trapped by Gb4-BSA and GbH-BSA, but not by BSA alone. Weaker associations between Rd-CSC HP-hP2 and BSA-Gb4 or BSA-GbH were suggested because more free forms of Rd-CSC HP-hP2 were monitored.

## DISCUSSION

Based on the development of the serum-free culture method to prepare CSCs, a series of CSC HPs were screened by a method composed of a negative selection with CCs and positive selection with CSCs. In random conditions, the possibility of successively picking up the same items from a library of 10^9^ kinds of items is 10^-9^. After the selection procedures, CSC HP-1 happened 10 times among the 31 valid sequences, indicating that the selection procedures were highly effective. In the other case, CSC HP-hP1 occurred 17 times among the 20 valid sequences, further confirming the efficiency of the selection strategy. In 2012, up to 744 tumor homing peptides (THPs) that had been reported or patented were collected in the TumorHoPe database [64]. Among these THPs, 36 peptides contained the RGD motif, which specifically recognized the angiogenic blood vessel markers integrin αvβ3 and αvβ5 [57, 58] and 41 peptides that contained the NGR motif. This motif binds to aminopeptidase N (CD13) [59, 60], a tumor angiogenesis marker, as well. None of the 15 CSC HPs reported in this study matched to the 744 THPs, simply because of different panning strategies. The strategies of THPs isolation were based on the differences either between cancer cells and normal cells or between tumor blood vessel and normal blood vessel. On the other hand, the strategy of CSC HPs screening was based on the differences between cancer stem cells and cancer cells. The vast cells in a tumor may be destroyed by THP-drug complex. The drug resistant CSCs can be further trapped by CSC HPs.

It is reasonable to picture that the distribution patterns of CSC surface markers on various types of CSCs are different. To confirm this postulation, the primary CSC HP-library was selected directly once by either CT26 or Hepa1-6 CSCs. As illustrated by the data shown in Table 1, CSC HP-8 was the most attractive for CT26, and CSC HP-3 was exclusively selected by Hepa1-6 CSCs. The bias of CSC HP affinities between CSCs and embryonic stem cells (ESCs) was another interesting issue. The CSC HP-1 was further enriched in the ESC bound fraction (CSC/ESC-HP-library), indicating that the target of CSC HP-1 may be a general stem cell surface marker. Two new peptides, CSC HP-9 and CSC HP-12, were read in the ESC unbound fraction (CSC HP-library) only.

SSEA-1, SSEA-3, SSEA-4, SSEA-5, GbH, and Le^Y^ pluripotent stem cell markers are the oligo-saccharide moieties of glycosphingolipids that are composed of complex glycans linked to sphingosines and various fatty acid chains [68–70]. The sphingosine and fatty acid part is also named as ceramide (Cer). According to the first hexose attached to the 1-hydroxyl of Cer via a β-glycosidic bond, mammalian glycosphingolipids can be divided into glucose- and galactose-subfamilies [71]. The galactose-subfamily (GalCer) and its 3-O-sulfated derivatives are predominant glycans in the brain. The glucose-subfamily (GlcCer) is further categorized into the Globo (Gb), Isoglobo (iGb), Lacto (Lc), Neolacto (nLc), and Ganglio (Gg) series according to the first tetrasaccharide root structures. SSEA-3 (Gb5), SSEA-4, and GbH are members of the Gb series. SSEA-1 and Le^Y^ are members of the nLc and SSEA-5 is a member of Lc series (Supplementary Figure 2A). During the early stage of mouse embryonic development, the Gb-series member SSEA3/4 is expressed at first with maximum at 4-cell stage. Then the nLc-series member SSEA-1 is maximally expressed at 8-32 cell morula stage followed by Le^Y^ of the same series at early blastula stage. The members of Gg-series are detected after implantation [66, 67].

According to the glycan microarray data, CSC HP-1 and CSC HP-hP1 bound strongly and specifically to glycan 26, which is the Globo root structure of tetrasaccharides. It can be concluded that, corresponding to the library screening data, CSC HP-1 and CSC HP-hP1 bind to general stem cell surface markers. On the other hand, CSC HP-5, CSC HP-6, CSC HP-7, and CSC HP9 bound strongly to glycan 19, which is the core trisaccharide of Lacto and Neolacto root structure tetrasaccharides. The CSC HP-3 and CSC HP-8, speculated as being specific to liver and colon CSCs, respectively, were found weakly bound to glycan 26. The real targets of CSC HP-3 and CSC HP-8 remain to be identified. CSC HP-1 and CSC HP-hP1 were the two CSC HP members that bound more strongly to glycan 63, Le^Y^ epitope, while the affinities were moderate only.

In addition to the well-known glycan moieties of stem cell markers, a new oligo-saccharide, glycan 69 (Neu5Ac-α-2,6-Gal-β-1,3-(Neu5Ac-α-2,6)-GalNAc-β-), was found to be strongly associated with the CSC HP-9, CSC HP-10, CSC HP-11, CSC HP-hP2, and CSC HP-hP3. Corresponding to the results that the two Neu5Ac-α-2,6- residues were necessary for these CSC HPs binding (Figure 5E), the enzymes possibly catalyzing the sialylation reactions, ST6Gal1 [72] and ST6GalNAc5 [73], were found to be highly expressed in malignant and metastatic tumors and corresponding to a worse prognosis [74–76]. Our western blotting data illustrated that both ST6Gal1 and ST6GalNAc5 were expressed in PANC-1 CSCs but not in PANC-1 CC (Figure 6B). Further studies are still needed to determine whether glycan 69 can be considered as a novel CSC marker.

In addition to the PANC-1 CSCs that could be bound by CSC HP-hP1-DsRed recombinant protein, a minority of the CCs were also stained by the recombinant protein (Figure 4B). This result indicates that PANC-1 cells cultured in serum-containing medium were heterogeneous as suggested by the stem cell model of cancer [77, 78]. These CSCs could be selectively amplified in the defined serum-free medium in suspension state. The CSCs in the tumorospheres, even expressing much higher levels of stem cell markers, should be heterogeneous as well. The linkage of the fluorescent dye to the N-terminus or the DsRed protein to the C-terminus of CSC HP-hP1 did not block its interactions with CSCs. These results indicate that the CSC HPs are potential vehicles to deliver anti-CSC reagents, either large or small molecules.

## MATERIALS AND METHODS

### Cancer cell and cancer stem cell cultures

Recombinant growth factors were purchased from PeproTech. Culture media and nutrient supplements were obtained from Invitrogen. Chemicals were acquired from Sigma-Aldrich. Mouse breast cancer EMT6, liver cancer Hepa1-6 and human pancreatic ductal adenoma PANC-1 cell lines were maintained in DMEM supplemented with 10% FCS. Mouse colon cancer CT26 and human lung cancer cell line H1650 were cultured in RPMI1640 supplemented with 10% FCS. Human colon cancer cell line HT29 was maintained in McCoy’s 5A complete medium supplemented with 10% FCS. The basic culture medium for CSCs was DMEM/F12 with 2 mM glutamine and the supplements for EMT6 CSCs were 25 ng/mL rmEGF, 25 ng/mL rmFGF2, 5 μg/mL insulin, 4 μg/mL heparin, 0.5 μg/mL hydrocortisone, and 1% BSA; for CT26 they were 20 ng/mL rmEGF, 5 μg/mL insulin, 2% B27, and 0.4% BSA; for Hepa1-6 that were 20 ng/mL rmEGF, 10 ng/mL rmFGF2, 2% B27, and1% N2; for PANC-1 they were 2% B27 and 20 ng/mL rhFGF2; for HT29 they were 20 ng/mL rhEGF, 5 μg/mL insulin, 2% B27, and 0.4% BSA; and for H1650 they were 20 ng/mL rhEGF, 10 ng/mL rhFGF2, 1% ITS, 10 μg/mL putrescine, 2 μM progesterone, and 0.4% BSA. In order to prepare tumorospheres, cancer cells were cultured in the serum-free media at low density, < 10^4^/mL. After 7 days of incubation, the tumorospheres were harvested using a 40 μm cell strainer, and they were centrifuged for 5 min at 900 × g at RT. The pellets of the tumorospheres were dissociated to single cells by trypsin, and then the obtained cells were expanded into tumorospheres again for another 7 days. Growth factors were replenished every second day, and tumorospheres isolated in the third to fifth round of expansion were used for experiments.

### M13 phage display

The M13 PhD-7 library (NEB, E8100) which contains 1×10^9^ independent clones was purchased from New England Biolabs Inc. 1×10^13^ plaque firming units (pfus) of M13 PhD-7 phages in 10 mL DMEM were pre-adsorbed by cancer cells in a T75 flask twice, each for 1 hour. Then the supernatant was transferred to a T25 flask containing 5×10^6^ CSCs and incubated for 1 hour. The unbound and weakly associated phages were removed by centrifugation and suspension three times in DMEM/0/2% BSA. The cell pellet was suspended in 1 mL PBS containing 1×10^9^ *E. coli* ER2738 and incubated in a shaker for 1 hour. Then, 10 mL LB/5 mM MgCl_2_ was added. After being cultured over-night at 37°C, the cells were removed by centrifugation and phages in the supernatant were titrated. The aforementioned selection procedures were carried out for three rounds for EMT6 (Figure 2A) and PANC-1 (Figure 2B) to obtain the primary EMT6 CSC HP- and PANC-1 CSC HP-library, respectively. Then, M13 plaques were picked up to prepare the DNA for sequencing. The EMT6 CSC HP-library was further selected by CT26 CSCs, Hepa1-6 CSCs, and mouse embryonic stem cells ESC (mESCs) again to prepare the secondary CT26 CSC HP-, Hepa1-6 CSC HP-, and CSC/ES HP-library. The unbound fraction after mESC adsorption was collected and as signed as the CSC PH-library (Figure 2A).

### Glycan microarray analysis

The N-terminus biotin conjugated CSC HPs were synthesized by GeneDirex Inc. with purity higher than 98% and were listed in Table 2. Glycan Array 100 microchips (Cat. # GA-Glycan-100) were purchased from RayBiotech and were processed following the manufacturer’s instruction with some modifications. Briefly, after blocking with 400 μL Sample Diluent (Item E) at room temperature for 30 min, the biotin conjugated CSC HPs were diluted to 1 μg/400 μL with Sample Diluent, and hybridization reactions were carried out at room temperature for 2 hours. After washing, 400 μL of 1× Cy3-conjugated streptavidin was added to each well. The incubation chamber was covered with the plastic adhesive strips and cover the slide with aluminum foil to avoid exposure to light during incubating in a dark room. The slide was incubated with Cy3-conjugated streptavidin at RT for 1 hour with gentle rocking or shaking. After thorough washing, the slide was drained completely, and the fluorescent signals on it were read by laser scanner Axon GenePix. The relative intensity on glycan X was defined as (average of X – negative control)/(positive control – negative control) × 100%.

### IF assay

The primary antibodies, rabbit polyclonal antibodies against CD44 (GTX102111), E-cadherin (GTX61823) and CDSN (GTX110093) as well as mouse monoclonal antibodies against SSEA-1 (GTX48038), SSEA-5 (GTX70019), and Lewis Y (GTX75903), were purchased from GeneTex; mouse monoclonal antibodies against SSEA-4 (SCR001 Part No. 90231), TRA-1-60 (SCR001 Part No. 90232) and TRA-1-81 (SCR001 Part No. 90233) were from Merck-Millipore; mouse monoclonal antibody against GbH (IgM, ALX-804-550) was from Enzo. The secondary antibodies DyLight594-conjugated goat anti-rabbit IgG (111-515-144) and DyLight488-conjugated donkey anti-mouse IgG (715-485-151) were obtained from Jackson Lab.; DyLight594-conjugated goat anti-mouse IgM (GTX76754) was obtained from GeneTex. The antibodies used in this report were summarized in Supplementary Table 1. Cancer cells and tumorospheres were fixed by 4% paraformaldehyde/PBS. Then the cells were incubated with primary antibodies that were diluted in TBS/1%BSA (25 mM Tris-HCl, pH 7.4/150 mM NaCl/1% BSA) by 50 to 100 fold for two hours. After 4 times washes with TBS (25 mM Tris-HCl, pH 7.4/150 mM NaCl), the cells were incubated with secondary antibodies (1/200 dilution in TBS/1%BSA) for 2 hours. After 4 washes with TBS and one brief wash with water, the cells were mounted with ProLong^®^ Gold Antifade Mount with DAPI (Invitrogen, P36935).

### Western blotting assay

The primary antibodies, rabbit polyclonal antibodies against ST6GalNAc5 (GTX45949), ST6Gal1 (GTX104018) and β-actin (GTX109369), and secondary HRP-conjugated goat anti-rabbit antibody were purchased from GeneTex. Each 100 μg of total lysate proteins from PANC-1 CCs or CSCs were separated by SDS PAGE before transferred onto a PVDF membrane. The primary antibodies against ST6GalNAc5, ST6Gal1 and β-actin were diluted by 1000, 1000 and 5000 folds, respectively, in blocking solution (20 mM Tris-HCl, pH 7.5/150 mM NaCl/3% non-fat milk). After incubation with the primary antibodies for 2 hours, the membranes were washed with wash solution (20 mM Tris-HCl, pH 7.5/150 mM NaCl/0.05% Tween-20) for 4 times. Then the membranes were incubated with the secondary antibody which was diluted in blocking solution for 2500 folds for 2 hours and washed for 4 times. ECL HRP substrate (Thermo-Fisher, 34075) was used to perform the chemiluminescent signals and images were taken with a CCD imaging system.

### Recombinant protein preparation

Supplementary Figure 1 shows the map of pET-CSC HP-hP1-DsRed vector. The *E.coli* Rosetta gamiB(DE3)pLysS host cells transformed by pET-CSC HP-hP1-DsRed were grown in 2×YT supplemented with 0.4% glucose, 30 μg/mL chloramphenicol, and 50 μg/mL ampicillin at 37°C. IPTG was adjusted to 1 mM when OD_600_ was 0.6 and cells were cultured for another 4 h. Then cells were collected by centrifugation at 10,000 *g* for 10 min. After ultra-sonication, the soluble form of CSC HP-hP1-DsRed recombinant protein was purified by using the Ni-Sepharose 6 Fast Flow (17-5318-02, GE) affinity column following the manufacturer’s instructions.

### Association between CSC HPs and CSCs

The N-terminus rhodamine- or fluorescein-conjugated CSC HPs were synthesized by GeneDirex Inc. with purity higher than 98% and were listed in Table 2. Cancer cells and tumorospheres fixed by 4% paraformaldehyde/PBS were incubated with 50 μg/mL of the conjugated peptides in TBS/1%BSA for an hour. After 3 times washes with TBS and a brief wash with water, the cells were mounted with ProLong^®^ Gold Antifade Mount with DAPI (Invitrogen, P36935).

### Association between CSC HPs and oligo-saccharides

Gb4-conjugated BSA (GLY121-BSA) and GbH-conjugated BSA (GLY123-BSA) were purchased from ELICITYL. The final concentrations of Rd-conjugated CSC HP-1, CSC HP-9, CSC HP-hP1, or CSC HP-hP2 were adjusted to 10 μg/mL and of BSA, Gb4-BSA or GbH-BSA were adjusted to 700 μg/mL in TNTD (10 mM Tris-HCl, pH 7.4/50 mM NaCl/0.02% Tween 20/0.02% sodium deoxycholate). After incubation for 30 minutes, the free Rd-CSC HPs were separated from those associated with BSA, Gb4-BSA or GbH-BSA by ultra-filtration. Amicon Ultra-0.5 tubes (Merck-Millipore, UFC501024, 10 kDa cutoff) were pre-run with 0.5 mL of blocking solution (10 mM Tris-HCl, pH 7.4/50 mM NaCl/0.2% Tween 20/0.2% sodium deoxycholate) to prevent non-specific adhesions of Rd-CSC HPs onto the filter. Each 0.25 mL of Rd-CSC HPs/Gb-BSA mixtures were loaded to the inner tubes. Centrifugation was set at 5000 rpm. The volume of filtrate was checked every 5 minutes. When about half of the samples were filtrated, each 100 μL of the filtrate was transferred to a well of a black 96-well plate. The fluorescent data were detected with a reader, FLUOstar Omega (BMG LABTECH), by setting the excitation wave length at 544 nm and emission wave length at 620 nm.

## SUPPLEMENTARY MATERIALS FIGURES AND TABLE



## Abbreviations

ABC: ATP-binding cassette
CC: cancer cell
CSC: cancer stem cell
EMT: epithelial-mesenchymal transition
MTS: multicellular tumor spheroids
SCID: severe combined immune-deficient
SSEA: stage-specific embryonic antigen
ST6Gal1: β-galactoside α-2,6-sialyltransferase 1
ST6GalNAc5: N-acetylgalactosaminide α-2,6-sialyltransferase 5
THP: tumor homing peptide
TIC: tumor initiating cell
