# Supplementary material for: A set of cancer stem cell homing peptides associating with the glycan moieties of glycosphingolipids
Source: Oncotarget. 2018 Apr 17;9(29):20490–507. doi: 10.18632/oncotarget.24960 (PMC5945507; doi:10.18632/oncotarget.24960)
Supplement: Supplementary file 1 [file oncotarget-09-20490-s001.pdf]

A set of cancer stem cell homing peptides associating with the glycan moieties of glycosphingolipids

SUPPLEMENTARY MATERIALS

pET-CSC HP hP1-DsRed

```
                                  T7 primer
                                  ------------------->
AGATCT CGATCCCCGCGAAAT TAATACGACTCACTAT AGG GGAATTGTGAGCGGATAACAATTCC CC TCTAGA AATAATTTTGTTT
BglII                     T7 promoter                   lac operator                 XbaI

                              NdeI
      AACTTTAAGAAGGAG ATATA CAT ATG GGT CCG AAA GTG ACC ATT TGG GGT GGA GGT
                 rbs              Met Gly Pro Lys Val Thr Ile Trp Gly Gly Gly

 BamHI    EcoRI        NdeI                                                XhoI
GGG ATC CCG AAT TCT (CATATG ATG GAC AAC DsRed TCC CAG GGT GGT) CTC GAG
Gly Ile Pro Asn Ser His Met Met Asp Asn ===== Ser Gln Gly Gly  Leu Glu

CAC CAC CAC CAC CAC CAC TGA GATCCGGCTGCTAACAAAGCCCGAAAGGAAGCTGAGTTGGCTGCTGCCACCGCTGAGCAATAA
His His His His His His stop
        His-tag

CTAGCATAACCCCTTGGGGCCTCTAAACGGGTCTTGAGGGGTTTTTTG
             T7 terminator
```

Supplementary Figure 1: Representative map of pET-CSC HP-hP-DsRed expressing vector. The expression cassette is derived by a T7 promoter. The coding region is composed of a CSC HP-hP1 peptide in front of a DsRed protein.

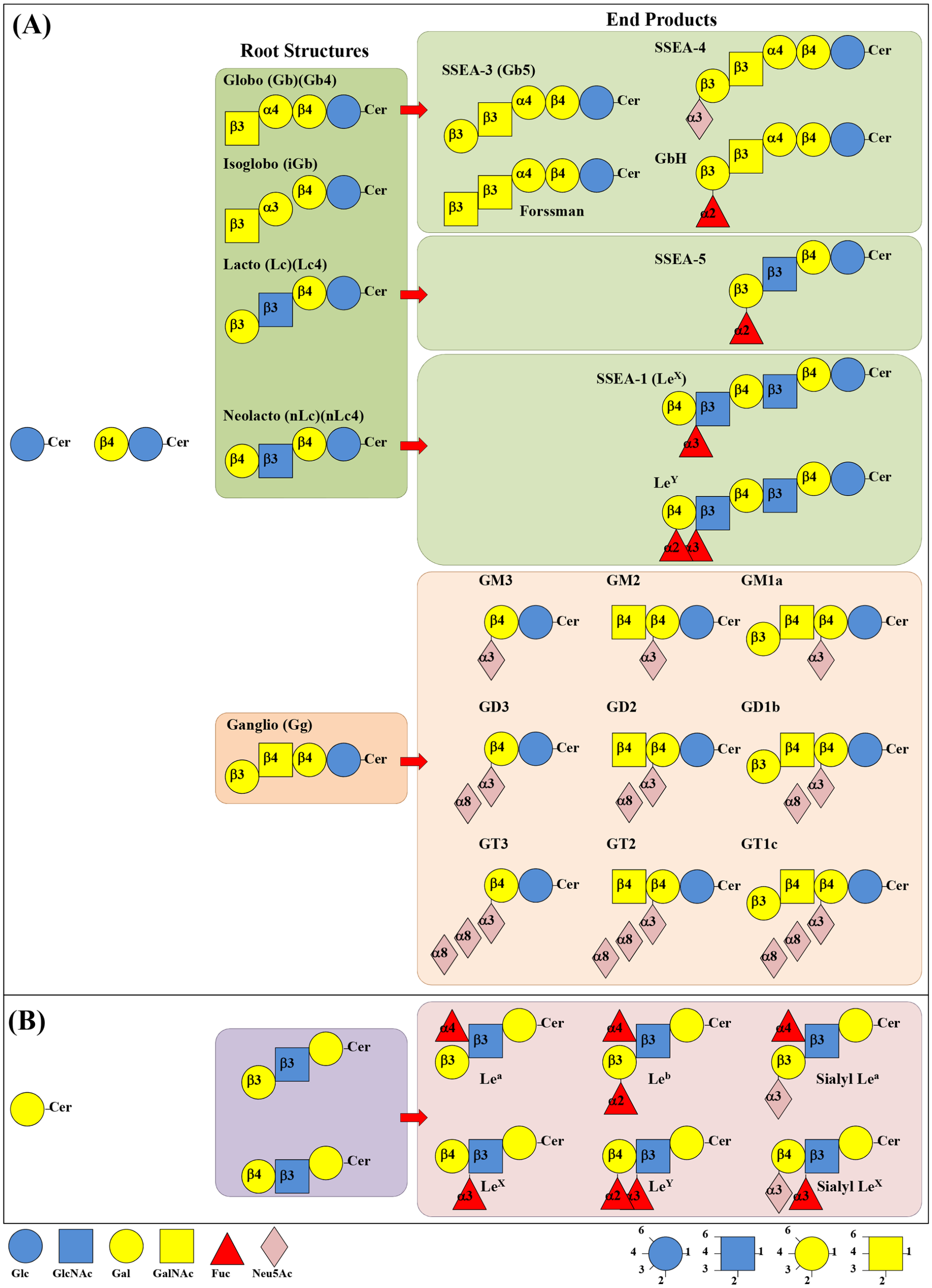

Supplementary Figure 2: Categories of the glycan groups on glycosphingolopids. (A) The glucose-subfamily (GlcCer) can be categorized into the Globo (Gb), Isoglobo (iGb), Lacto (Lc), Neolacto (nLc), and Ganglio (Gg) series according to the first tetrasaccharide root structures. SSEA-3 (Gb5), SSEA-4, and GbH are members of the Gb series. SSEA-1 and Le^{Y} are members of the nLc and SSEA-5 is a member of Lc series. **(B)** The member of galactose-subfamily (GalCer) are highly fucosylated and/or sialylated and are predominant glycans in the brain. The symbols for glucose N-acetylglucosamine, galactose, N-caetylgalactosamine, fucose, and 5-acetylneuraminic acid as well as the numbering of carbon are shown in the bottom.

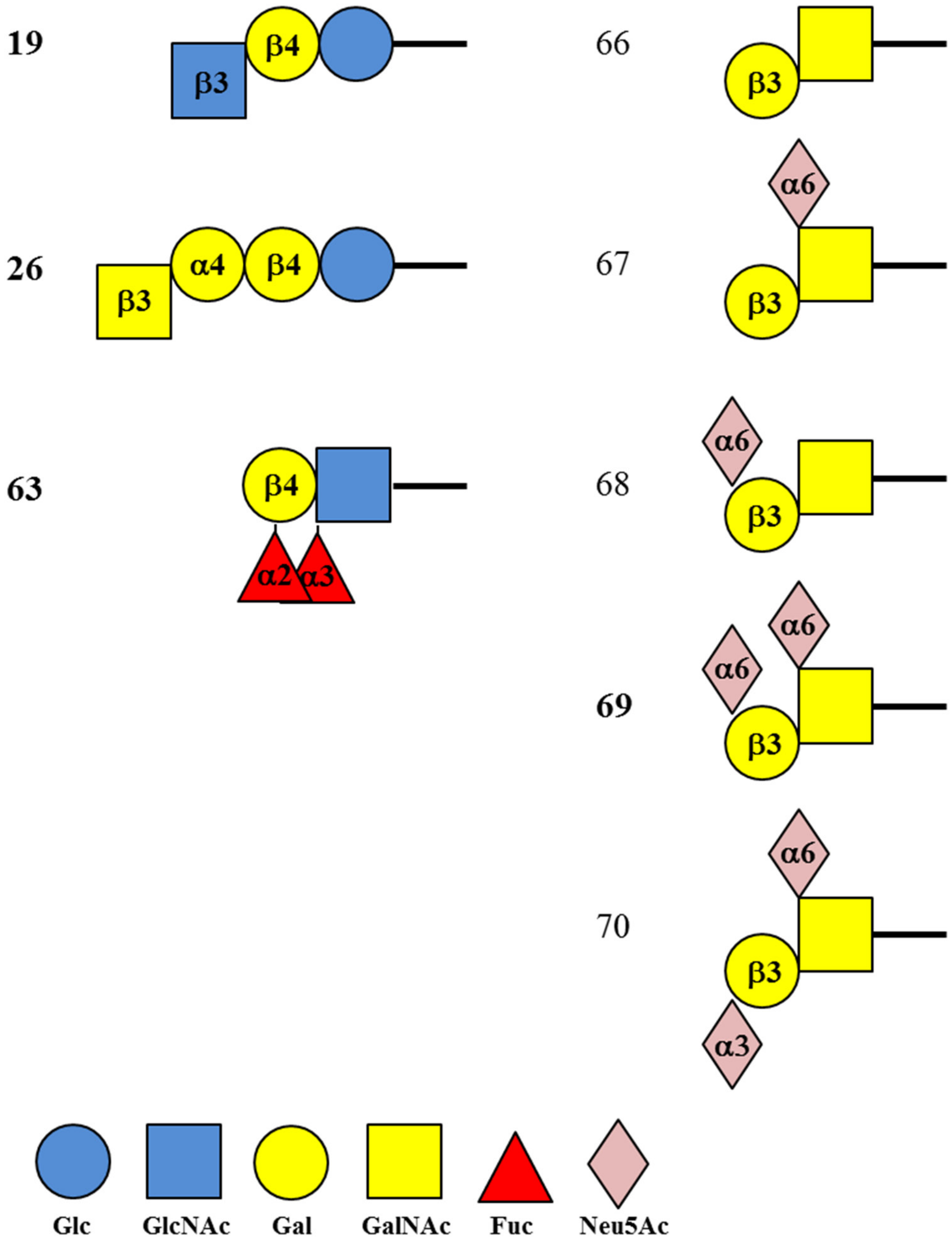

Supplementary Figure 3: Structures of the Glycans interacted with CSC HPs spotted on the Glycan Array 100 microchip. The structures of glycan 19, 26, 63, 66, 67, 68 ,69, and 70 are as the symbols described above.

Supplementary Table 1: Primary and secondary antibodies used in this study

Marker	Primary antibody	Secondary antibody
**CD44**	Rabbit polyclonal Abs (GeneTex, GTX102111)	DyLight594-conjugated goat anti-rabbit IgG (Jackson, 111-515-144)
**CD133**	Rabbit polyclonal Abs (GeneTex, GTX102109)	DyLight594-conjugated goat anti-rabbit IgG (Jackson, 111-515-144)
**E-cadherin**	Rabbit polyclonal Abs (GeneTex, GTX61823)	DyLight594-conjugated goat anti-rabbit IgG (Jackson, 111-515-144)
**SSEA-1**	Mouse mAb (IgM) (GeneTex, GTX48038)	DyLight594-conjugated goat anti-mouse IgM (GeneTex, GTX76754)
**SSEA-4**	Mouse mAb (IgG) (Millipore, SCR001 Part No. 90231)	DyLight488-conjugated donkey anti-mouse IgG (Jackson, 715-485-151)
**SSEA-5**	Mouse mAb (IgG) (GeneTex, GTX70019)	DyLight488-conjugated donkey anti-mouse IgG (Jackson, 715-485-151)
**GbH**	Mouse mAb (IgM) (Enzo, ALX-804-550)	DyLight594-conjugated goat anti-mouse IgM (GeneTex, GTX76754)
**Lewis Y**	Mouse mAb (IgM) (GeneTex, GTX75903)	DyLight594-conjugated goat anti-mouse IgM (GeneTex, GTX76754)
**TRA-1-60**	Mouse mAb (IgM) (Millipore, SCR001 Part No. 90232)	DyLight594-conjugated goat anti-mouse IgM (GeneTex, GTX76754)
**TRA-1-81**	Mouse mAb (IgM) (Millipore, SCR001 Part No. 90233)	DyLight594-conjugated goat anti-mouse IgM (GeneTex, GTX76754)
**CDSN**	Rabbit polyclonal Abs (GeneTex, GTX110093)	DyLight594-conjugated goat anti-rabbit IgG (Jackson, 111-515-144)
**ST6GalNAc5**	Rabbit polyclonal Abs (GeneTex, GTX45949)	Goat anti-rabbit IgG antibody (HRP) (GeneTex, GTX213110-01)
**ST6Gal1**	Rabbit polyclonal Abs (GeneTex, GTX104018)	Goat anti-rabbit IgG antibody (HRP) (GeneTex, GTX213110-01)
**β-actin**	Rabbit polyclonal Abs (GeneTex, GTX109639)	Goat anti-rabbit IgG antibody (HRP) (GeneTex, GTX213110-01)
